# Regioselective Synthesis of 6-*O*-Acetyl Dieckol and Its Selective Cytotoxicity against Non-Small-Cell Lung Cancer Cells

**DOI:** 10.3390/md20110683

**Published:** 2022-10-29

**Authors:** Hyeon-Cheol Shin, Yongkyun Kim, Jaeyeong Choi, Hyun Bae Kang, Seung-Yun Han, Kwangyong Park, Hye Jeong Hwang

**Affiliations:** 1 Center for Molecular Intelligence, The State University of New York, Korea, 119 Songdomunhwa-ro, Yeonsu-gu, Incheon 21985, Korea; 2 School of Chemical Engineering and Material Science, Chung-Ang University, 84 Heukseok-ro, Dongjak-gu, Seoul 03722, Korea; 3 Healinnols Inc., 1662 Yuseong-daero, Yuseong-gu, Daejeon 34054, Korea; 4 College of Medicine, Konyang University, 158 Gwanjeodong-ro, Seo-gu, Daejeon 35365, Korea

**Keywords:** dieckol, regiospecific modification, cytotoxicity, selective index, non-small-lung cancer cell

## Abstract

Dieckol, a phlorotannin from *Ecklonia cava*, has shown potential for use as an anticancer agent that selectively kills cancer cells. However, it is necessary to amplify its potency without damaging its inherent safety in order to develop it as a competitive chemotherapeutic. Here, we explored the controlled *O*-acylations of dieckol. Acyl groups could be consistently introduced to the 6-*O* position of dieckol with a high regioselectivity, which was confirmed by NOESY, HMBC and HSQC spectroscopies. In cytotoxicity studies on the newly synthesized 6-*O*-acetyl, 6-*O*-benzoyl dieckols and previously synthesized 6-*O*-alkyl dieckols against A549 vs. normal cells, all of the derivatives showed low cytotoxicity in normal cells with an IC_50_ of 481–719 μM, and highly structure-dependent cytotoxicity in A549 cells with an IC_50_ of 7.02 (acetyl)−842.26 (benzyl) μM. The selectivity index also showed a large structure dependency in the range of 0.67 (benzyl)–68.58 (acetyl). An analysis of the structure–activity relationship indicated that the activity was dramatically reduced in the presence of a benzene ring and was highly increased in the presence of small polar substituents. Conclusions: Controlled mono-*O*-modifications of dieckol could be a powerful tool to enhance the anticancer activity of dieckol, thus contributing to the development strategy for dieckol-based chemotherapeutics.

## 1. Introduction

Typically, anticancer agents are notorious for their serious adverse effects that negatively impact the different organs of cancer patients, which ultimately aggravate their suffering [1]. In traditional small-molecular anticancer chemotherapy, which are based on the fact that cancer cells tend to grow faster than normal cells, the drugs travel throughout the body and damage normal, healthy cells that are also fast-growing, causing various side effects [2]. The normal cells that are most likely to be damaged by chemotherapies are blood-forming cells in the bone marrow, hair follicles, cells in the mouth, digestive tract and the reproductive system. Some chemo drugs can damage cells in the heart, kidneys, bladder, lungs and the nervous system [3]. 

In contrast to the small molecules, anticancer antibodies are more targeted toward cancer signaling pathways and exhibit fewer side effects than traditional small-molecular chemotherapy. However, their interference with the immune system which triggers serious inflammation- and infection-related adverse effects as well as their extremely high cost limit their use. Furthermore, most of these drugs have very narrow therapeutic windows, making it difficult to render an effective therapeutic dose without experiencing toxic effects [1]. Therefore, it is highly necessary to explore a novel class of small molecules derived from the understudied pool of marine natural products that may have extraordinary anticancer selectivity. 

A special class of phlorotannins called “eckols”, which are characterized by a dibenzo-*p*-dioxin molecular skeleton [4], are found in several types of brown algae and have recently gained attention for their wide spectrum of pharmacological activities such as antioxidant, anti-inflammatory, antidiabetic, hepatoprotective, neuroprotective, radioprotective, matrix metalloproteinase inhibitory, anticoagulative, antibacterial, antiviral, anti-obesity, antihistamine and antihypertensive activities [5]. A standardized form of their natural extract had been approved as an investigational new drug (IND) from the US FDA and is currently being developed as a botanical drug candidate for diabetic complications [6,7]. Some previous studies have shown promise for various modes of anticancer therapies such as anti-carcinogenesis [8], chemo-sensitization [9], anti-angiogenesis [10], anti-metastasis [11,12,13] and anti-stemness [14]. Among the various eckols, dieckol (DK, **1**) isolated from *Ecklonia cava* has recently been shown to induce the apoptosis of ovarian cancer cells and to inhibit tumor xenograft growth without side effects [15], indicating its great potential as a novel class of chemotherapeutic agent that selectively kills cancer cells over normal cells.

However, it is necessary to amplify the anticancer activity and optimize the pharmacological characteristics of DK to overcome its natural scarcity and develop it as a real-world, competitive pharmaceutical. To address this issue, it is crucial both to maximize the activity and to optimize its physicochemical properties including the polarity, p*K*a, solubility and metabolic stability, via a controlled structural modification of DK, while retaining its inherent safety in normal cells. 

Regarding a modification site in DK, eleven aromatic C−H carbons and another eleven phenolic oxygen atoms can be theoretically considered. However, a modification of the C−H bond would be disadvantageous for two reasons. Firstly, a controlled and high-yield modification at the aromatic C−H bond in DK would be both extremely difficult and far from a green chemistry because it would require the regioselective activation of the very strong C−H bond, which requires the use of a transition-metal catalyst. Secondly, modifications at the carbon atom would profoundly impact the biochemical nature of the dibenzo-*p*-dioxin skeleton of DK, leading to the loss of its essential merits in pharmacological and metabolic aspects. 

On the other hand, in the case of *O*-modifications, where the dibenzo-*p*-dioxin skeleton is intact, it is very feasible for the synthetic derivatives to inherit the original biochemical characteristics of DK including its pharmacological activities and metabolic fate, thereby minimizing the risk of increased toxicity and side effects. Furthermore, in synthetic aspects, it is advantageous to utilize the nucleophilicity of the phenolic oxygens to efficiently introduce an unlimited variety of chemical groups. However, even in this scenario, it is still very challenging to introduce a substituent to a specific oxygen atom out of eleven virtually equivalent ones with a high yield.

So far, a very limited amount of information has been obtained on the controlled introduction of substituents to DK. Kwak et al. reported the study on the mono-*O*-propargylation of dieckol and subsequent click reaction using the propargyl group to conjugate DK with fluorescent moieties [16,17]. More recently, we reported the first general synthetic method to prepare several mono-alkylated DKs via a regioselective reaction at a specific phenolic position of DK [18]. In that study, some of the DK derivatives showed slightly enhanced cytotoxicity (1.4–2.6 times) against a breast cancer cell line (BT-20). Although the study showed the first proof-of-concept for the controlled derivatization of DK, the modification was limited to alkylation alone, and the impact of the modifications on the important issue, i.e., selectivity against cancer cells vs. normal cells, was not addressed. 

Acylation is another feasible reaction that can be considered to modify the phenolic -OHs in DK. However, concerning the acylation of DK or other phlorotannins, those only reported were per-acetylation of phlorotannins to avoid the oxidation of air-sensitive polyphenolic molecules during the purification process for analytical purposes [19,20]. Therefore, there has not been any study on the controlled acylation of DK within the scope of our knowledge. 

In our current study, we aimed to expand the knowledge on the controlled *O*-modification of DK for the anticancer arena in the following three ways: (1) How can we introduce various acyl groups to DK in a controlled manner? (2) What kind of *O*-substituents would significantly enhance the cytotoxicity against cancer cells? and (3) What kind of *O*-substituents would be desirable to maximize the selectivity in cytotoxicity against cancer vs. normal cells? 

To address these questions, we investigated various reaction parameters of acylation, determined the exact site of acylation via intensive 2D-NMR analyses and measured the cytotoxicity of the newly synthesized *O*-acyl DK derivatives together with the previously synthesized *O*-alkyl DK derivatives against non-small-cell lung cancer (NSCLC) and normal cells. 

## 2. Results

### 2.1. Acylation Reactions of Dieckol (***1***)

**Scheme 1:**
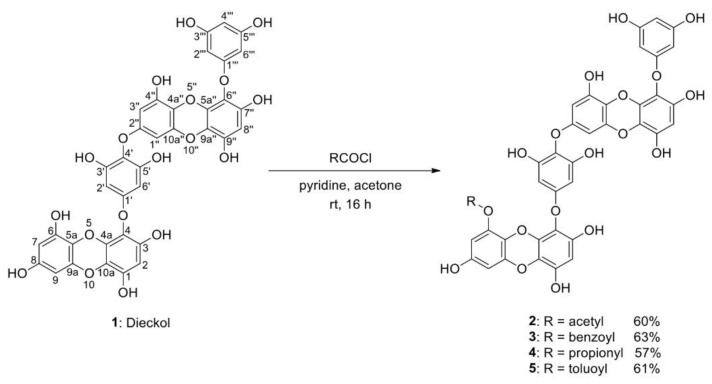
Regioselective mono-O-acylation of dieckol (**1**).

First, we conducted the *O*-acetylation of dieckol by varying the base, solvent, temperature, reaction time, and type and concentration of the acetylating agents. Acetyl chloride and acetic anhydride were investigated as the acetylating agents, and K_2_CO_3_, pyridine and triethylamine were examined as bases. Among a variety of reaction conditions, the reaction of DK with 1 equiv. of acetyl chloride in the presence of 1 equiv. of pyridine in dry acetone for 16 h at 25 °C gave the highest yield of the mono-substituted adduct (mono-*O*-acetyl dieckol (**2**)), which was obtained in 60% isolated yield as a pale-yellow powder. Consequently, these parameters were determined to be the optimal reaction conditions for the mono-*O*-acylation of DK (Scheme 1). At this reaction condition, a cluster of minor products with a lower polarity than that of the compound **2** occurred (isolated yield of 15%), which was speculated to be a mixture of many differently acetylated DK derivatives based on the complex nature of the peaks in ^1^H-NMR spectroscopy. By integrating the hydroxyl protons at δ 9.3−9.7, nine hydroxyl protons out of the eleven hydroxyl protons in DK were revealed to be present, indicating it to be a mixture of various di-acetylated adducts of DK (data not shown).

*O*-benzoyldieckol (**3**), *O*-propionyldieckol (**4**), and *O*-*p*-toluoyldieckol (**5**) were also prepared under similar optimized reaction conditions. All of the acylation reactions proceeded smoothly to afford the corresponding substituted products as a pale-yellow powder in 57–63% isolated yield. The minor products that occurred under the reaction conditions also showed a similar pattern to the case of acetylation (data not shown).

### 2.2. Spectroscopic Determination of Exact Structures of O-Acyl Dieckols

In order to confirm the exact structures, especially the exact positions of the substitution of the acylated DKs, intensive NMR spectroscopic analyses were performed. The ^1^H-NMR spectrum of **2** showed a methyl resonance peak at δ = 2.02 (s, 3H) (Table 1), thereby confirming the successful introduction of an acetyl group. Peaks corresponding to 10 hydroxyl groups were detected in the NMR spectrum at δ = 9.67 (s, C_8_-OH), 9.65 (s, C_1_-OH), 9.63 (s, C_4″_-OH), 9.42 (s, C_9″_-OH), 9.38 (s, C_3_-OH), 9.31 (s, C_3′_-OH and C_5′_-OH), 9.19 (s, C_7″_-OH) and 9.12 (s, C_3__‴_-OH and C_5__‴_-OH) ppm. The ^13^C NMR spectrum also confirmed the introduction of a single acetyl group, as evident from the two singlets at δ = 168.1 and 20.2 ppm, corresponding to a carbonyl carbon and a methyl carbon, respectively. Although the ^1^H and ^13^C NMR spectra clearly indicated the regioselective formation of an *O*-acetyl-dieckol regioisomer as the major product, they did not provide enough information for determining the exact position of the acetyl group on the product. Therefore, detailed 2D NMR studies were necessary to elucidate the exact structure of the product.

**Figure 1:**
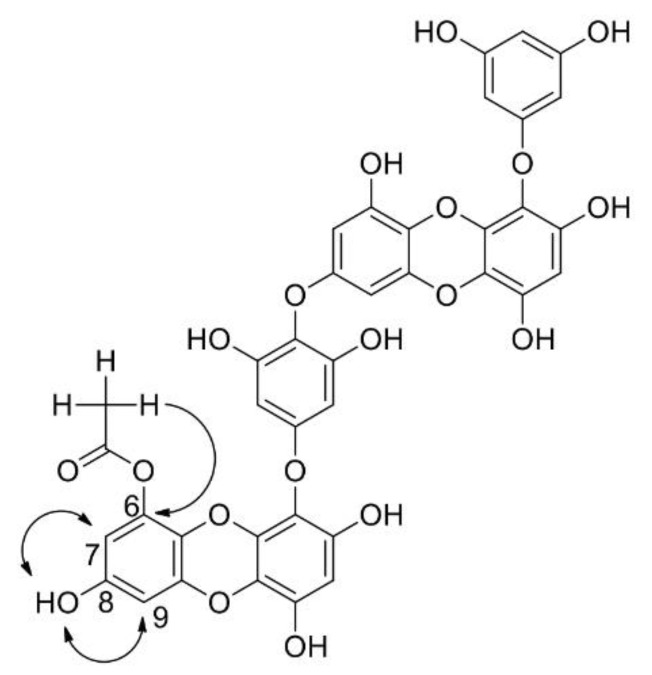
Key HMBC (H to C, →) and NOESY (H to H, ↔) correlations of **2**.

**Figure 2:**
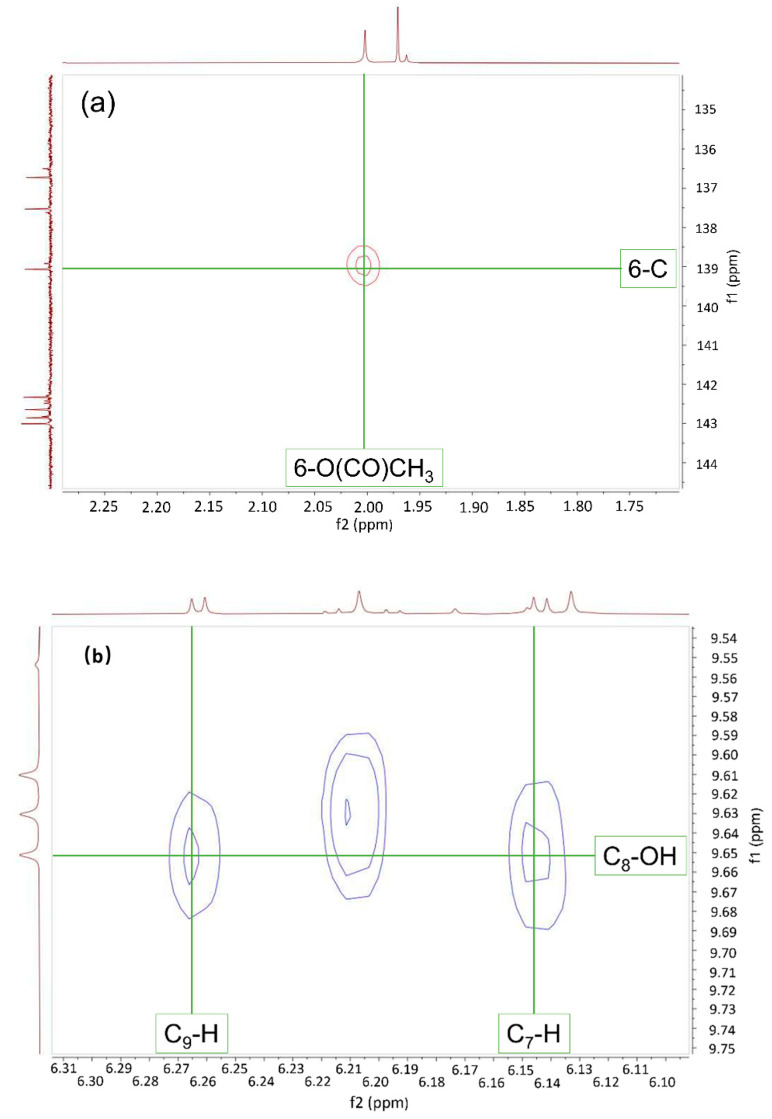
Key HMBC (**a**) and NOESY (**b**) spectra of **2**.

The following correlations between the aromatic proton and carbon were obtained from the HSQC spectra: δ_H_ 6.22–δ_C_ 99.04 (C_2_), δ_H_ 6.16–δ_C_ 104.91 (C_7_), δ_H_ 6.28–δ_C_ 101.01 (C_9_), δ_H_ 5.92–δ_C_ 94.32 (C_2′_, C_6′_), δ_H_ 5.81–δ_C_ 93.72 (C_1″_), δ_H_ 6.06–δ_C_ 98.81 (C_3″_), δ_H_ 6.15–δ_C_ 98.73 (C_8″_), δ_H_ 5.80–δ_C_ 96.63 (C_4__‴_) and δ_H_ 5.73–δ_C_ 94.07 (C_2__‴_, C_6__‴_). The connectivity of all of the protons and carbons was elucidated by HMBC spectroscopy. In the HMBC spectrum, the acetyl protons at δ 2.02 showed a correlation with the aromatic carbon at δ 139.05, indicating the attachment of an *O*-acetyl moiety at the 6-*C* position of dieckol (Figure 1 and Figure 2a).

The structure of **2** was further assessed by NOESY experiments. Unfortunately, the expected nuclear Overhauser effect (NOE) correlation of the 6-*O*-acetyl group with C_7_-H was not observed in the NOESY spectrum. However, the presence of the C_8_-OH (δ_H_ 9.67)/C_7_-H (δ_H_ 6.16) and C_8_-OH (δ_H_ 9.67)/C_9_-H (δ_H_ 6.28) correlations with the absence of the C_6_-OH/C_7_-H correlation clearly indicated that substitution occurred at the C_6_-*O* position (Figure 1 and Figure 2b). All of the HMBC and NOESY correlations are listed in Table 1.

The structures of compounds **3**, **4** and **5** could also be confirmed by thorough analysis of the ^1^H and ^13^C NMR spectra (Table 2). For example, the structure of 6-*O*-benzoyl dieckol (**3**) was also determined from the 2D NMR studies. However, unlike the case of 6-*O*-acetyl dieckol, the presence of the benzoyl group on C_6_ could not be directly identified because of the absence of an appropriate proton in the benzoyl moiety that could correlate with C_6_ through HMBC interactions. The key NOE correlations, namely C_8_-OH (δ_H_ 9.76)/C_9_-H (δ_H_ 6.35) and C_8_-OH (δ_H_ 9.76)/C_7_-H (δ_H_ 6.38), with the absence of the C_6_-OH/C_7_-H correlation in the NOESY spectrum confirmed that the benzoyl group was introduced at the 6-position (Table S1).

### 2.3. Cytotoxicity

**Figure 3:**
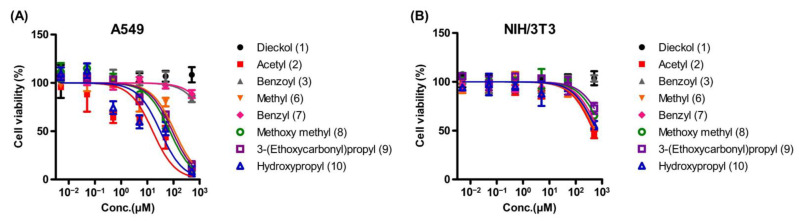
Cytotoxicity trends of DK derivatives. (**A**) against A549 NSCLC cells and (**B**) normal (NIH/3T3) cells. Data are expressed as mean values ± SD from three separate experiments.

The cytotoxicity of DK and its various acyl and alkyl derivatives was assessed using the MTT assay against a non-small-lung cancer cell line (A549) and a normal cell line (NIH/3T3) at six different concentrations (0, 0.005, 0.05, 0.5, 5, 50 and 500 μM). Among the newly synthesized acyl derivatives, the 6-*O*-acetyl (**2**) and 6-*O*-benzoyl (**3**) derivatives were included in this study, while the 6-*O*-propionyl (**4**) and 6-*O*-toluoyl (**5**) derivatives, which had been considered to be very similar to 6-*O*-acetyl (**2**) and 6-*O*-benzoyl (**3**) derivatives, respectively, were excluded from this study. As alkyl derivatives, five previously synthesized 6-*O*-alkyl derivatives (compounds **6**–**10**)^18^ were included in the study. The methyl and benzyl derivatives (**6** and **7**) were selected in parallel with acetyl and benzoyl (**2** and **3**) derivatives, respectively, to identify any impact of substitution chemistry (via sp^2^ vs. sp^3^ carbon in acylations and alkylations, respectively) and steric effect in cytotoxicity. Methoxymethyl (**8**), 3-(ethoxycarbonyl)propyl (**9**) and hydroxypropyl (**10**) derivatives were selected to determine the size effect of moderately polar substituents with a common heteroatom such as oxygen. The results are summarized in Figure 3 and Table 3.

DK did not show detectable cytotoxicity within the studied concentration range in either of the cell lines studied. On the contrary, all of the DK derivatives except 6-*O*-benzyl DK exhibited cytotoxicity in both cell lines. While the DK derivatives generally showed a low level of cytotoxicity with the IC_50_ range of 481.42−719.30 μM against the normal cell line, they showed a highly substituent-dependent cytotoxicity (IC_50_ 7.02−842.26 μM) against the A549 cancer cell line with the following order of potency: Acetyl > Hydroxypropyl > Methoxymethyl > 3-(Ethoxycarbonyl)propyl > Methyl >> Benzoyl > Benzyl. 

In the assessment of the selective cytotoxicity against A549 cells vs. normal cells, the DK derivatives also showed a large substituent dependency in the range of 0.67 (Benzyl) −68.58 (Acetyl) with the following order: Acetyl > Hydroxypropyl > Methoxymethyl > 3-(Ethoxycarbonyl)propyl > Methyl >> Benzyl.

## 3. Discussion

Four acyl groups including acetyl and benzoyl groups were efficiently introduced at the C_6_–*O* position of dieckol (**1**) with remarkably high regioselectivity and good yields, despite the presence of 10 other nearly equivalent *O*-substitution sites (phenolic oxygens) in DK. Initially, it was expected that C_1_–*O*, C_8_–*O*, C_3__‴_–*O* and C_5__‴_–*O*, which are at the most sterically open positions, would preferentially attack the electrophilic carbonyl carbons of acyl reagents, especially with benzoyl chloride, which is much bulkier than acetyl chloride. However, the substitutions occurred predominantly at the more congested C_6_–*O* position, regardless of the size of the acylating reagent (e.g., 60% and 63% yield at the C_6_–*O* position for acetyl and benzoyl, respectively). The same regioselectivity also occurred in the alkylation of DK [18]. Considering that all of the acylation and alkylation reactions were performed under weak base-catalyzed aprotic polar solvent systems, the observed regioselectivity at the C_6_–*O* position indicates that the nucleophilicity of oxygen at the C_6_ position was the highest under this reaction condition. 

However, it is also very surprising that C_4″_–*O*, whose chemical environment is almost identical to that of C_6_–*O*, did not participate in the reaction, within the limits of our analysis. One explanation for this could be as follows. Since the lowest p*K*a of DK is estimated to be 5.0 [21], the basicity of pyridine would be strong enough to almost completely deprotonate the most acidic hydroxyl group in DK. Based on the consistent reaction at C_6_–*O*, it is presumed that the C_6_–*OH* is the most acidic and preferentially deprotonated to reveal a much more nucleophilic phenoxide ion that would readily attack the electrophilic carbons in acyl and alkyl halides. Since all of the atoms in DK are speculated to be electronically connected with each other, at least partially, through π–π (aromatic system) or π–*p*–π (C^aromatic^–O–C^aromatic^) conjugations [21], once the C_6_–*OH* is deprotonated first, when the negative charge on DK would contribute to a significant increase in p*K*a of the second most acidic OH, then the same base would not be basic enough to deprotonate any other OH on DK, so that the nucleophilic substitution reaction would happen predominantly on C_6_–*OH*. If this is the case, it would be possible to enhance the regioselectivity further by optimizing the base. However, further elaborate kinetic and theoretical investigations together with meticulous analyses of minor products are required to understand the accurate origin of such remarkable difference in the reactivity of the seemingly equivalent phenolic hydroxyl groups at different positions of DK. Aside from academic curiosity regarding this huge separation of reactivity in the two OH groups in C_6_ and C_4”_, in a practical sense, such a remarkable regioselectivity in the *O*-substitution of DK as discovered in this study can be utilized to develop and optimize a variety of pharmaceutically and pharmacologically prominent DK-based drug candidates. 

The controlled introduction of non-functional alkyl or acyl groups such as methyl, benzyl, acetyl and benzoyl moiety to DK would be useful in fine-tuning the pharmacological parameters such as solubility, pharmacokinetic and/or pharmacodynamic profiles of the potential DK-based drug candidates. On the other hand, the introduction of a protection group such as methoxymethyl (MOM) to the C_6_–*O* position would open a door to the controlled introduction of a substituent at other positions of DK than C_6_–*O*. Furthermore, controlled introductions of active functional groups such as carbonyl and alcohol groups (compound **9** and **10**) could be used for controlled conjugations of DK with a variety of small molecules or proteins, etc. 

Lung cancer is the leading cause of cancer death worldwide [22]. Lung cancer has been historically divided into two main types: non-small-cell lung cancer (NSCLC) and small-cell lung cancer (SCLC). SCLC is a malignant tumor that accounts for about 15% of lung cancer, with NSCLC accounting for the remaining 85% [23]. 

In the cytotoxicity study against the A549 NSCLC cell line, all of the studied 6-*O*-acyl and 6-*O*-alkyl DKs showed some cytotoxicity, while DK did not. The cytotoxicity of the derivatives showed a heavy dependency on the chemical nature of the substituents (120-fold difference between the highest and lowest potent derivatives). The presence of a benzene ring on the substituent at the C_6_–*O* position dramatically decreased the cytotoxicity against A549 cells, as shown in benzyl and benzoyl DK with a 7-fold and 91-fold increase in IC_50_ compared with methyl and acetyl DK, respectively. The introduction of a substituent via *sp*^2^ (via acylation) or *sp*^3^ (via alkylation) carbon did not significantly influence such tendency, as shown in the very high IC_50_ in both the benzyl and benzoyl DKs. 

**Figure 4:**
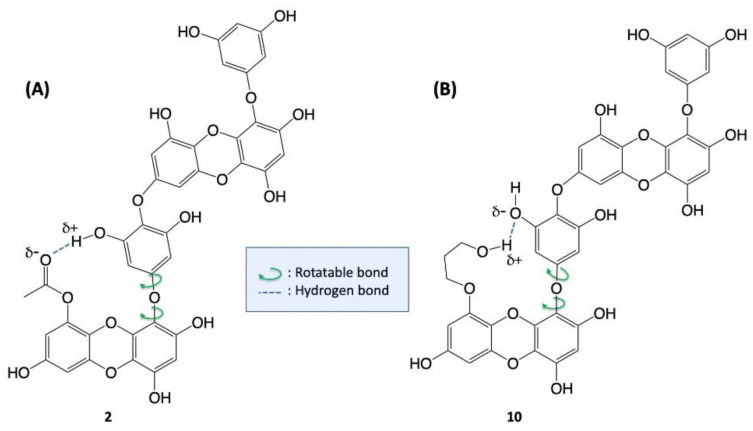
Speculated effect of the small polar substituents at the C_6_–*O* position on the conformational isomerism of DK. Compounds **2** and **10** showed the highest activities. The two rotatable bonds can participate in the conformational isomerism of DK depending on the environment of the phloroglucinol ring. (**A**) Possible attractive force via H-bonding between the partially negative carbonyl oxygen in compound **2** and the hydroxyl group in the nearby phloroglucinol ring. (**B**) Possible attractive force via H-bonding between the terminal hydroxyl group of compound **10** and the hydroxyl group in the nearby phloroglucinol ring.

On the other hand, small and polar substituents tended to show higher activity, as shown in the three derivatives with the lowest IC_50_ such as acetyl, hydroxypropyl and methoxymethyl DKs, while methyl DK with the smallest but nonpolar substituent showed the highest IC_50_ except for benzyl and benzoyl DKs. It is worth mentioning here that DK is an oligomer (hexamer) of phloroglucinol which has six single bonds that can be involved in conformational isomerism [24]. Therefore, it is presumed that the presence of a small polar substituent such as an acetyl or hydroxypropyl group at the C_6_–*O* position may have a hydrogen bonding interaction with a hydroxyl group in the nearby phloroglucinol ring (Figure 4) to induce the DK’s conformation to be more favorable for anti-A549 cytotoxicity [25]. 

On the contrary, it is also presumed that the presence of a rigid and bulky benzene ring at C_6_–*O* may sterically repel the nearby phloroglucinol ring to induce the DK’s conformation to be unfavorable for anti-A549 cytotoxicity. However, it is necessary to perform elaborate theoretical calculations of each derivative under an aqueous environment to confirm the exact driving force for such a significant substituent dependence, which warrants a dedicated study. 

The ideal anticancer drug should have a relatively high toxic concentration on normal cells with a very low active concentration on cancer cells [26]. In this study, the cancer-selective cytotoxicity of DK derivatives has been studied for the first time. To address this selectivity issue, the selectivity index (SI), which is defined as the ratio of the toxic concentration of a sample against its effective bioactive concentration according to Equation (1) (see Section 4.7), was used [27]. In this study, two DK derivatives, 6-*O*-acetyl (68.58) and 6-*O*-hydroxypropyl (43.07) DKs, showed SI values > 10. An SI value ≥ 10 was assumed to belong to a selected potential sample that can be further investigated [28]. Therefore, the acetyl and hydroxypropyl DKs would be highly considered for further investigations as potential anti-NSCLC chemotherapeutic agents.

## 4. Materials and Methods

### 4.1. Materials

Dieckol (**1**, 98.5%) was isolated from Ecklonia cava collected off the coast of Jeju Island, South Korea and supplied by Botamedi Inc. (Jeju, Korea). Benzoyl chloride and *p*-toluoyl chloride were purchased from Sigma-Aldrich Co. (St. Louis, MO, USA). Acetyl chloride, pyridine, acetone, chloroform and methanol were purchased from Daejung Chemicals (Gyeonggi-do, Republic of Korea). Propionyl chloride was purchased from Alfa Aesar (Ward Hill, MA, USA). Acetone was dehydrated with 4 Å molecular sieves before use. Analytical thin-layer chromatography (TLC) was performed using Merck Kieselgel 60 F_254_ pre-coated plates (0.25 mm) with a fluorescent indicator and visualized under UV light (254 and 365 nm). Column chromatography was performed on silica gel 60, 70−230 mesh. Dulbecco’s Modified Eagle Medium (DMEM), fetal bovine serum (FBS), and penicillin–streptomycin were obtained from Gibco Co. (Carlsbad, CA, USA). Lastly, 3-(4,5-dimethythiazol-2-yl)-2,5-diphenyltetrazolium bromide (MTT) was purchased from TCI Inc. (Tokyo, Japan).

### 4.2. Analytical Methods

^1^H NMR (600 MHz) and ^13^C NMR (150 MHz) were acquired on Varian NMR system 600 MHz spectrometers (VNS, Varian, Palo Alto, CA, USA) using DMSO-*d_6_* as a solvent. Chemical shifts were referenced to the residual solvent peaks (*δ_H_* 2.50 and *δ_C_* 39.5 for DMSO-*d_6_* in ^1^H NMR and ^13^C NMR, respectively). All coupling constants, *J*, are reported in hertz (Hz). 

### 4.3. Preparation of Mono O-Acyl DKs

#### 4.3.1. General Method of Preparation

Dry acetone (100 mL) was added to a mixture of dieckol (**1**, 300 mg, 0.404 mmol) and 32.6 μL (0.404 mmol) of pyridine. After stirring for 10 min, acyl chloride (0.404 mmol) was added in small portions. The reaction mixture was stirred at 25 °C for 16 h, diluted with EtOAc (200 mL), and washed successively with 1% aqueous HCl, water and saturated aqueous NaCl. Thereafter, the organic layer was dried over MgSO_4_, filtered and concentrated *in vacuo*. The crude compound was purified by column chromatography (MeOH: CHCl_3_ = 1:40 to 1:10) to afford the corresponding acyl DK (**2**–**5**) as a pale yellow powder. 

#### 4.3.2. Acetyl DK (**2**)

Dieckol (**1**, 300 mg, 0.404 mmol) was reacted with acetyl chloride (28.7 μL, 0.404 mmol) in the presence of pyridine (32.6 μL, 0.404 mmol) to afford **2** (190.2 mg, 60%): TLC *R_f_* = 0.34 (CHCl_3_: MeOH: H_2_O = 60:30:4, *v*/*v*/*v*); ^1^H NMR (600 MHz, DMSO-*d_6_*) *δ* 9.67 (s, 1H, C_8_-OH), 9.65 (s, 1H, C_1_-OH), 9.63 (s, 1H, C_4″_-OH), 9.42 (s, 1H, C_9″_-OH), 9.38 (s, 1H, C_3_-OH), 9.31 (s, 2H, C_3′,5′_-OH), 9.19 (s, 1H, C_7″_-OH), 9.12 (s, 2H, C_3__‴,5__‴_-OH), 6.28 (d, *J* = 2.73 Hz, 1H, C_9_-H), 6.22 (s, 1H, C_2_-H), 6.16 (d, *J* = 2.73 Hz, 1H, C_7_-H), 6.15 (s, 1H, C_8″_-H), 6.06 (d, *J* = 2.86 Hz, 1H, C_3″_-H), 5.92 (s, 2H, C_2′,6′_-H), 5.81 (d, *J* = 2.86 Hz, 1H, C_1″_-H), 5.80 (t, *J* = 2.08 Hz, 1H, C_4__‴_-H), 5.73 (d, *J* = 2.08 Hz, 2H, C_2__‴,6__‴_-H), 2.02 (s, 3H, C_6_-OCO*CH_3_*); ^13^C NMR (150 MHz, DMSO-*d_6_*) *δ* 168.11 (s, 1C, C_6_-O*(CO)*CH_3_), 160.70 (s, 1C, C_1__‴_), 159.20 (s, 2C, C_3__‴,5__‴_), 156.12 (s, 1C, C_1′_), 154.68 (s, 1C, C_2″_), 153.52 (s, 1C, C_8_), 151.66 (s, 2C, C_3′,5′_), 146.85 (s, 1C, C_3_), 146.46 (s, 1C, C_7″_), 146.33 (s, 1C, C_4″_), 142.99 (s, 1C, C_9a_), 142.84 (s, 1C, C_10a″_), 142.61 (s, 1C, C_1_), 142.32 (s, 1C, C_9″_), 139.05 (s, 1C, C_6_), 137.51 (s, 1C, C_5a″_), 136.71 (s, 1C, C_4a_), 126.36 (s, 1C, C_5a_), 124.63 (s, 1C, C_4′_), 124.52 (s, 1C, C_4a″_), 123.58 (s, 1C, C_9a″_), 123.56 (s, 1C, C_10a_), 122.61 (s, 1C, C_6″_), 122.30 (s, 1C, C_4_), 104.91 (s, 1C, C_7_), 101.01 (s, 1C, C_9_), 99.04 (s, 1C, C_2_), 98.81 (s, 1C, C_3″_), 98.73 (s, 1C, C_8″_), 96.63 (s, 1C, C_4__‴_), 94.32 (s, 2C, C_2′,6′_), 94.07 (s, 2C, C_2__‴,6__‴_), 93.72 (s, 1C, C_1″_), 20.18 (s, 1C, C_6_-O(CO)*CH_3_*).

#### 4.3.3. Benzoyl DK (**3**) 

Dieckol (**1**, 300 mg, 0.404 mmol) was reacted with benzoyl chloride (46.9 μL, 0.404 mmol) in the presence of pyridine (32.6 μL, 0.404 mmol) to afford **3** (215.5 mg, 63%): TLC *R_f_* = 0.40 (CHCl_3_: MeOH: H_2_O = 60:30:4, *v*/*v*/*v*); ^1^H NMR (600 MHz, DMSO-*d_6_*) *δ* 9.76 (s, 1H, C_8_-OH), 9.67 (s, 1H, C_1_-OH), 9.65 (s, 1H, C_4″_-OH), 9.41 (s, 1H, C_9″_-OH), 9.34 (s, 1H, C_3_-OH), 9.19 (s, 1H, C_7″_-OH), 9.17 (s, 2H, C_3′,5′_-OH), 9.12 (s, 2H, C_3__‴,5__‴_-OH), 7.81 (dd, *J_1_* = 7.04 Hz, *J_2_* = 1.32 Hz, 2H, C_6_-O(CO)C*(CH)_2_*(CH)_2_(CH)), 7.69 (td, *J_1_* = 7.47 Hz, *J_2_* = 1.32 Hz, 1H, C_6_-O(CO)C(CH)_2_(CH)_2_*(CH)*), 7.50 (dd, *J_1_* = 7.47 Hz, *J_2_* = 7.04 Hz, 2H, C_6_-O(CO)C(CH)_2_*(CH)_2_*(CH)), 6.38 (d, *J* = 2.73 Hz, 1H, C_7_-H), 6.35 (d, *J* = 2.73 Hz, 1H, C_9_-H), 6.23 (s, 1H, C_2_-H), 6.15 (s, 1H, C_8″_-H), 6.06 (d, *J* = 2.85 Hz, 1H, C_3″_-H), 5.80 (t, *J* = 2.09 Hz, 1H, C_4__‴_-H), 5.78 (d, *J* = 2.85 Hz, 1H, C_1″_-H), 5.73 (d, *J* = 2.09 Hz, 2H, C_2__‴,6__‴_-H), 5.70 (s, 2H, C_2′,6′_-H); ^13^C NMR (150 MHz, DMSO-*d_6_*) *δ* 164.01 (s, 1C, C_6_-O(CO)C(CH)2(CH)2(CH)), 160.70 (s, 1C, C_1__‴_), 159.20 (s, 2C, C_3__‴,5__‴_), 155.79 (s, 1C, C_1′_), 154.66 (s, 1C, C_2″_), 153.55 (s, 1C, C_8_), 151.41 (s, 2C, C_3′,5′_), 146.76 (s, 1C, C_3_), 146.46 (s, 1C, C_7″_), 146.33 (s, 1C, C_4″_), 143.16 (s, 1C, C_9a_), 142.80 (s, 1C, C_10a″_), 142.65 (s, 1C, C_1_), 142.31 (s, 1C, C_9″_), 139.07 (s, 1C, C_6_), 137.48 (s, 1C, C_5a″_), 137.05 (s, 1C, C_4a_), 134.31 (s, 1C, C_6_-O(CO)*C*(CH)_2_(CH)_2_(CH)), 130.02 (s, 2C, C_6_-O(CO)C*(CH)_2_*(CH)_2_(CH)), 129.91 (s, 2C, C_6_-O(CO)C(CH)_2_*(CH)_2_*(CH)), 128.54 (s, 1C, C_6_-O(CO)C(CH)_2_(CH)_2_*(CH)*), 126.47 (s, 1C, C_5a_), 124.61 (s, 1C, C_4′_), 124.50 (s, 1C, C_4a″_), 123.58 (s, 1C, C_9a″_), 123.52 (s, 1C, C_10a_), 122.60 (s, 1C, C_6″_), 122.14 (s, 1C, C_4_), 105.03 (s, 1C, C_7_), 101.13 (s, 1C, C_9_), 99.21 (s, 1C, C_2_), 98.85 (s, 1C, C_3″_), 98.73 (s, 1C, C_8″_), 96.62 (s, 1C, C_4__‴_), 94.20 (s, 2C, C_2′,6′_), 94.07 (s, 2C, C_2__‴,6__‴_), 93.83 (s, 1C, C_1″_).

#### 4.3.4. Propionyl DK (**4**)

Dieckol (**1**, 300 mg, 0.404 mmol) was reacted with propionyl chloride (35.1 μL, 0.404 mmol) in the presence of pyridine (32.6 μL, 0.404 mmol) to afford **4** (186.2 mg, 57%): TLC *R_f_* = 0.34 (CHCl_3_: MeOH: H_2_O = 60:30:4, *v*/*v*/*v*); ^1^H NMR (600 MHz, DMSO-*d_6_*) *δ* 9.66 (s, 1H, C_8_-OH), 9.65 (s, 1H, C_1_-OH), 9.64 (s, 1H, C_4″_-OH), 9.44 (s, 1H, C_9″_-OH), 9.37 (s, 1H, C_3_-OH), 9.30 (s, 2H, C_3′,5′_-OH), 9.18 (s, 1H, C_7″_-OH), 9.12 (s, 2H, C_3__‴,5__‴_-OH), 6.28 (d, *J* = 2.71 Hz, 1H, C_9_-H), 6.22 (s, 1H, C_2_-H), 6.16 (d, *J* = 2.71 Hz, 1H, C_7_-H), 6.15 (s, 1H, C_8″_-H), 6.05 (d, *J* = 2.87 Hz, 1H, C_3″_-H), 5.90 (s, 2H, C_2′,6′_-H), 5.81 (d, *J* = 2.87 Hz, 1H, C_1″_-H), 5.80 (t, *J* = 2.11 Hz, 1H, C_4__‴_-H), 5.73 (d, *J* = 2.11 Hz, 2H, C_2__‴,6__‴_-H), 2.36 (q, *J* = 7.48, 2H, C_6_-O(CO)*CH_2_*CH_3_), 0.99 (t, *J* = 7.48, 3H, C_6_-O(CO)CH_2_*CH_3_*); ^13^C NMR (150 MHz, DMSO-*d_6_*) *δ* 171.25 (s, 1C, C_6_-O*(CO)*CH_2_CH_3_), 160.72 (s, 1C, C_1__‴_), 159.21 (s, 2C, C_3__‴,5__‴_), 156.04 (s, 1C, C_1′_), 154.67 (s, 1C, C_2″_), 153.53 (s, 1C, C_8_), 151.65 (s, 2C, C_3′,5′_), 146.80 (s, 1C, C_3_), 146.47 (s, 1C, C_7″_), 146.34 (s, 1C, C_4″_), 143.00 (s, 1C, C_9a_), 142.84 (s, 1C, C_10a″_), 142.64 (s, 1C, C_1_), 142.33 (s, 1C, C_9″_), 139.16 (s, 1C, C_6_), 137.52 (s, 1C, C_5a″_), 136.81 (s, 1C, C_4a_), 126.37 (s, 1C, C_5a_), 124.62 (s, 1C, C_4′_), 124.52 (s, 1C, C_4a″_), 123.60 (s, 1C, C_9a″_), 123.53 (s, 1C, C_10a_), 122.63 (s, 1C, C_6″_), 122.23 (s, 1C, C_4_), 104.95 (s, 1C, C_7_), 100.96 (s, 1C, C_9_), 99.10 (s, 1C, C_2_), 98.83 (s, 1C, C_3″_), 98.74 (s, 1C, C_8″_), 96.63 (s, 1C, C_4__‴_), 94.22 (s, 2C, C_2′,6′_), 94.09 (s, 2C, C_2__‴,6__‴_), 93.73 (s, 1C, C_1″_), 26.67 (s, 1C, C_6_-O(CO)*CH_2_*CH_3_), 9.07 (s, 1C, C_6_-O(CO)CH_2_*CH_3_*).

#### 4.3.5. Toluoyl DK (**5**)

Dieckol (**1**, 300 mg, 0.404 mmol) was reacted with *p*-toluoyl chloride (53.4 μL, 0.404 mmol) in the presence of pyridine (32.6 μL, 0.404 mmol) to afford **5** (211.4 mg, 61%): TLC *R_f_* = 0.40 (CHCl_3_: MeOH: H_2_O = 60:30:4, *v*/*v*/*v*); ^1^H NMR (600 MHz, DMSO-*d_6_*) *δ* 9.73 (s, 1H, C_8_-OH), 9.65 (s, 1H, C_1_-OH), 9.65 (s, 1H, C_4″_-OH), 9.41 (s, 1H, C_9″_-OH), 9.34 (s, 1H, C_3_-OH), 9.19 (s, 1H, C_7″_-OH), 9.16 (s, 2H, C_3′,5′_-OH), 9.12 (s, 2H, C_3__‴,5__‴_-OH), 7.71 (d, *J* = 8.05 Hz, 2H, C_6_-O(CO)C*(CH)_2_*(CH)_2_CCH_3_), 7.31 (d, *J* = 8.05 Hz, 2H, C_6_-O(CO)C(CH)_2_*(CH)_2_*CCH_3_), 6.37 (d, *J* = 2.74 Hz, 1H, C_7_-H), 6.34 (d, *J* = 2.74 Hz, 1H, C_9_-H), 6.23 (s, 1H, C_2_-H), 6.15 (s, 1H, C_8″_-H), 6.07 (d, *J* = 2.88 Hz, 1H, C_3″_-H), 5.81 (t, *J* = 2.10 Hz, 1H, C_4__‴_-H), 5.79 (d, *J* = 2.88 Hz, 1H, C_1″_-H), 5.73 (d, *J* = 2.10 Hz, 2H, C_2__‴,6__‴_-H), 5.69 (s, 2H, C_2′,6′_-H), 2.40 (3H, C_6_-O(CO)C(CH)_2_(CH)_2_C*CH_3_*); ^13^C NMR (150 MHz, DMSO-*d_6_*) *δ* 163.99 (s, 1C, C_6_-O*(CO)*C(CH)_2_(CH)_2_CCH_3_), 160.73 (s, 1C, C_1__‴_), 159.23 (s, 2C, C_3__‴,5__‴_), 155.81 (s, 1C, C_1′_), 154.70 (s, 1C, C_2″_), 153.53 (s, 1C, C_8_), 151.43 (s, 2C, C_3′,5′_), 146.78 (s, 1C, C_3_), 146.48 (s, 1C, C_7″_), 146.34 (s, 1C, C_4″_), 144.75 (s, 1C, C_6_-O(CO)C(CH)_2_(CH)_2_*C*CH_3_), 143.17 (s, 1C, C_9a_), 142.82 (s, 1C, C_10a″_), 142.66 (s, 1C, C_1_), 142.34 (s, 1C, C_9″_), 139.19 (s, 1C, C_6_), 137.51 (s, 1C, C_5a″_), 137.07 (s, 1C, C_4a_), 130.08 (s, 2C, C_6_-O(CO)C*(CH)_2_*(CH)_2_CCH_3_), 129.84 (s, 2C, C_6_-O(CO)C(CH)_2_*(CH)_2_*CCH_3_), 126.51 (s, 1C, C_5a_), 125.79 (s, 1C, C_6_-O(CO)*C*(CH)_2_(CH)_2_CCH_3_), 124.68 (s, 1C, C_4′_), 124.54 (s, 1C, C_4a″_), 123.62 (s, 1C, C_9a″_), 123.58 (s, 1C, C_10a_), 122.64 (s, 1C, C_6″_), 122.17 (s, 1C, C_4_), 105.07 (s, 1C, C_7_), 101.04 (s, 1C, C_9_), 99.19 (s, 1C, C_2_), 98.92 (s, 1C, C_3″_), 98.75 (s, 1C, C_8″_), 96.65 (s, 1C, C_4__‴_), 94.21 (s, 2C, C_2′,6′_), 94.10 (s, 2C, C_2__‴,6__‴_), 93.93 (s, 1C, C_1″_), 21.87 (s, 1C, C_6_-O(CO)C(CH)_2_(CH)_2_C*CH_3_*).

### 4.4. Preparation of Mono-O-Alkyl DKs

Mono *O*-alkyl DKs with methyl (**6**), benzyl (**7**), methoxymethyl (**8**), hydroxypropyl, 3-(Ethoxycarbonyl)propyl (**9**) and hydroxypropyl (**10**) substituents were prepared according to the method described earlier [18].

### 4.5. Cell Lines and Culture

The non-small-cell lung cancer cell line A549 and the normal cell line NIH/3T3 were purchased from Korea Cell Line Research Foundation (Seoul, Korea). The cell lines were cultured in Dulbecco’s modified Eagle’s medium (DMEM) supplemented with 10% FBS and 1% penicillin–streptomycin at 37 °C with 5% CO_2_. 

### 4.6. Cytotoxicity Assay

The cytotoxicity of DK and its derivatives was assessed by MTT assay, based on the reduction of MTT into insoluble formazan crystals by metabolically active cells. Briefly, the cells (5 × 10^5^) were seeded in each well containing culture medium in a 96-well plate. After the incubation for 24 h at 37 °C, various concentrations of DK and its derivatives were added. After 24 h, 10 μL of the MTT reagent (5 μg/mL) was added, and the plates were incubated for an additional 2 h. The medium was discarded, and the formazan blue, which was formed in the cells, was dissolved in 500 μL of dimethyl sulfoxide (DMSO). The optical density was measured at 540 nm using a microplate spectrophotometer (Epoch; Agilent Technologies, Inc. Santa Clara, CA, USA). The IC_50_ corresponded to a 50% decrease in the number of cells compared to the untreated control. The results are represented as the mean of three independent experiments. The IC_50_ values of DK and its derivatives were determined for the different cell lines by nonlinear regression using GraphPad Prism v6.0 software (La Jolla, CA, USA).

### 4.7. Selectivity Index

The selectivity index (SI) was determined to assess the selective cytotoxicity of the DK derivatives against NSCLC (A549) cells vs. normal (NIH/3T3) cells based on Equation (1):

SI = IC_50_ (NIH/3T3 cells)/IC_50_ (A549 cells) (1)

### 4.8. Statistical Analysis

The experimental data were shown as the mean ± SD for at least triplicate determination of three independent experiments. The obtained results were analyzed using one-way ANOVA and Tukey’s post hoc test, and *p*-values < 0.05 were considered statistically significant. 

## 5. Conclusions

The 6-*O*-acyl derivatives of DK were prepared in high yields with surprisingly high regioselectivity. The exact position of derivatization was confirmed by thorough 2D-NMR analyses using HMBC, HSQC and NOESY spectroscopies. It is the first report for controlled acylations of phlorotannins and DK. It was demonstrated that the anticancer activity of DK against A549 non-small-lung-cancer cell was greatly improved by introducing small polar groups such as acetyl and hydroxypropyl groups via either acylation or alkylation at the C_6_-*O* position of DK. Moreover, 6-*O*-acetyl DK showed 120 times higher activity than 6-*O*-benzyl DK.

The anticancer selectivity of DK derivatives has been studied for the first time. In the evaluation of the selective index for the cytotoxicity of the DK derivatives against A549 vs. normal cells, two compounds, acetyl and hydroxypropyl DKs, showed SI values of 68.58 and 43.07, respectively, warranting further investigation as anticancer drug leads. 

Therefore, the regioselective substitution at the C_6_-*O* position of DK with a range of chemical groups including various acyl and alkyl groups could be a powerful and versatile tool, not only to enhance the anticancer activity of DK, but also to fine-tune the pharmacological parameters and to conjugate DK with other chemical or biological drug molecules. 

## Figures and Tables

**Table 1:** ^1^H, ^13^C, HMBC and NOESY data of **2** (δ in ppm, data obtained in DMSO-*d_6_*).

No.	*δ*_C_	*δ*_H_	(*J* in Hz)	HMBC (H → C)	NOESY
**1**	142.61	9.65		C_2_, C_1_, C_10a_	C_2_−H
**2**	99.04	6.22		C_4_, C_3_, C_1_, C_10a_	C_3_−H, C_1_−H
**3**	146.85	9.38		C_4_, C_3_, C_2_	C_2_−H
**4**	122.30				
**4a**	136.71				
**5a**	126.36				
**6**	139.05				
**7**	104.91	6.16	d (2.74)	C_9_, C_8_, C_6_, C_5a_	C_8_−H
**8**	153.52	9.67		C_9_, C_8_, C_7_	C_9_−H, C_7_−H
**9**	101.01	6.28	d (2.74)	C_9a_, C_8_, C_7_, C_5a_	C_8_−H
**9a**	142.99				
**10a**	123.56				
**1′**	156.12				
**2′**	94.32	5.92		C_1′_, C_3′_, C_4′_, C_5′_, C_6′_	C_3′_−H
**3′**	151.66	9.31		C_2′_, C_3′_, C_4′_, C_5′_, C_6′_	C_2′_−H
**4′**	124.63				
**5′**	151.66	9.31		C_2′_, C_3′_, C_4′_, C_5′_, C_6′_	C_6′_−H
**6′**	94.32	5.92		C_1′_, C_2′_, C_3′_, C_4′_, C_5′_	C_5′_−H
**1″**	93.72	5.81	d (2.86)	C_10a″_, C_2″_, C_3″_, C_4a″_	
**2″**	154.68	-			
**3″**	98.81	6.06	d (2.86)	C_1″_, C_2″_, C_4″_, C_4a″_	C_4"_−H
**4″**	146.33	9.63		C_3″_, C_4″_, C_4a″_	C_3__″_−H
**4a″**	124.52				
**5a″**	137.51				
**6** **″**	122.61				
**7** **″**	146.46	9.19		C_6″_, C_7″_, C_8″_	C_8__″_−H
**8** **″**	98.73	6.15		C_6″_, C_7″_, C_9″_, C_9a″_	C_7__″_−H, C_9__″_−H
**9** **″**	142.32	9.42		C_8″_, C_9″_, C_9a″_	C_8__″_−H
**9a** **″**	123.58				
**10a** **″**	142.84				
**1‴**	160.70				
**2‴**	94.07	5.73	d ( 2.08 )	C_1‴_, C_3‴_, C_4‴_, C_5‴_, C_6‴_	C_3__‴_−H
**3‴**	159.20	9.12		C_2‴_, C_3‴_, C_4‴_, C_5‴_, C_6‴_	C_2__‴_−H, C_4__‴_−H
**4‴**	96.63	5.80	t ( 2.08 )	C_2‴_, C_3‴_, C_5‴_, C_6‴_	C_3__‴_−H, C_5__‴_−H
**5‴**	159.20	9.12		C_2‴_, C_3‴_, C_4‴_, C_5‴_, C_6‴_	C_4__‴_−H, C_6__‴_−H
**6‴**	94.07	5.73	d ( 2.08 )	C_1‴_, C_2‴_, C_3‴_, C_4‴_, C_5‴_	C_5__‴_−H
**6-O*(CO)*CH_3_**	168.11				
**6-O(CO)*CH_3_***	20.18	2.02		C_6_, C_6_-O*(CO)*CH_3_	C_2__′_−H, C_6__′_−H

**Table 2:** ^1^H-NMR and ^13^C-NMR data of **1**–**5** (δ in ppm, data obtained in DMSO-*d_6_*).

No.	1		2		3		4		5	
	*δ*_C_	*δ*_H_	*δ*_C_	*δ*_H_	*δ*_C_	*δ*_H_	*δ*_C_	*δ*_H_	*δ*_C_	*δ*_H_
**1**	142.37	9.47	142.61	9.65	142.64	9.65	142.65	9.67	142.66	9.65
**2**	98.63	6.16	99.04	6.22	99.10	6.22	99.21	6.23	99.19	6.23
**3**	146.31	9.25	146.85	9.38	146.80	9.37	146.76	9.34	146.78	9.34
**4**	122.68	-	122.30	-	122.23	-	122.14	-	122.17	-
**4a**	137.63	-	136.71	-	136.81	-	137.05	-	137.07	-
**5a**	122.99	-	126.36	-	126.37	-	126.47	-	126.51	-
**6**	146.45	9.57	139.05	-	139.16		139.07	-	139.19	-
**7**	98.90	5.99	104.91	6.16	104.95	6.16	105.03	6.38	105.07	6.37
**8**	153.47	9.19	153.52	9.67	153.53	9.66	153.55	9.76	153.53	9.73
**9**	94.27	5.81	101.01	6.28	100.96	6.28	101.13	6.35	101.04	6.34
**9a**	142.99	-	142.99	-	143.00	-	143.16	-	143.17	-
**10a**	123.63	-	123.56	-	123.53	-	123.52	-	123.58	-
**1′**	156.30	-	156.12	-	156.04	-	155.79	-	155.81	-
**2′**	94.90	5.95	94.32	5.92	94.22	5.90	94.20	5.70	94.21	5.69
**3′**	151.55	9.32	151.66	9.31	151.65	9.30	151.41	9.17	151.43	9.16
**4′**	124.62	-	124.63	-	124.62	-	124.61	-	124.68	-
**5′**	151.55	9.32	151.66	9.31	151.65	9.30	151.41	9.17	151.43	9.16
**6′**	94.90	5.95	94.32	5.92	94.22	5.90	94.20	5.70	94.21	5.69
**1″**	93.94	5.82	93.72	5.81	93.73	5.81	93.83	5.78	93.93	5.79
**2″**	154.63	-	154.68	-	154.67	-	154.66	-	154.70	-
**3″**	98.46	6.02	98.81	6.06	98.83	6.05	98.85	6.06	98.92	6.07
**4″**	146.36	9.67	146.33	9.63	146.34	9.64	146.33	9.65	146.34	9.65
**4a″**	124.42	-	124.52	-	124.52	-	124.50	-	124.54	-
**5a″**	137.46	-	137.51	-	137.52	-	137.48	-	137.51	-
**6″**	122.61	-	122.61	-	122.63	-	122.60	-	122.64	-
**7″**	146.49	9.20	146.46	9.19	146.47	9.18	146.46	9.19	146.48	9.19
**8″**	98.75	6.14	98.73	6.15	98.74	6.15	98.73	6.15	98.75	6.15
**9″**	142.27	9.42	142.32	9.42	142.33	9.44	142.31	9.41	142.34	9.41
**9a″**	123.55	-	123.58	-	123.60	-	123.58	-	123.62	-
**10a″**	142.80	-	142.84	-	142.84	-	142.80	-	142.82	-
**1‴**	160.69	-	160.70	-	160.72	-	160.70	-	160.73	-
**2‴**	94.05	5.72	94.07	5.73	94.09	5.73	94.07	5.73	94.10	5.73
**3‴**	159.20	9.13	159.20	9.12	159.21	9.12	159.20	9.12	159.23	9.12
**4‴**	96.63	5.80	96.63	5.80	96.63	5.80	96.62	5.80	96.65	5.81
**5‴**	159.20	9.13	159.20	9.12	159.21	9.12	159.20	9.12	159.23	9.12
**6‴**	94.05	5.72	94.07	5.73	94.09	5.73	94.07	5.73	94.10	5.73

**Table 3:** Substituent-dependent cytotoxicity of DK and its derivatives against A549 vs. NIH/3T3 cell lines.

Substituent	Cytotoxicity ^*b*^ (IC_50_^*a*^, μM)		SI Value ^*c*^
	A549	NIH/3T3	
Intact DK (**1**)	N/D	N/D	N/A
Acetyl (**2**)	7.02 ± 1.53	481.42 + 18.61	68.58
Benzoyl (**3**)	640.92 ± 17.86	N/D	N/A
Methyl (**6**)	123.02 ± 1.85	575.79 ± 22.32	4.68
Benzyl (**7**)	842.26 ± 10.80	563.42 ± 34.02	0.67
Methoxymethyl (**8**)	66.10 ± 8.47	653.45 ± 27.46	9.89
3-(Ethoxycarbonyl)propyl (**9**)	82.45 ± 6.49	719.30 ± 54.58	8.72
Hydroxypropyl (**10**)	13.07 ± 5.06	562.90 ± 51.77	43.07

*^a^* IC_50_ is defined as the concentration (μM) that results in a 50% decrease in the number of cells compared to that of the control cultures. *^b^* The values are expressed as mean ± SD. *^c^* Selectivity index (SI) was determined to assess the selective cytotoxicity of the DK derivatives against NSCLC (A549) cells vs. normal (NIH/3T3) cells.

## Data Availability

The original contributions presented in the study are included in the article; further inquiries can be directed to the corresponding authors.

## Supplementary Materials

The following supporting information can be downloaded at: https://www.mdpi.com/article/10.3390/md20110683/s1, Figure S1: Structures of **2**–**5**, Figures S2–S15: 1H, 13C, HSQC, HMBC, NOESY spectra of compounds **2**–**5**. Tables S1–S3: Complete NMR data of compounds **2**–**5**.
