# Supplementary material for: Regioselective Synthesis of 6-O-Acetyl Dieckol and Its Selective Cytotoxicity against Non-Small-Cell Lung Cancer Cells
Source: Mar Drugs. 2022 Oct 29;20(11):683. doi: 10.3390/md20110683 (PMC9695823; doi:10.3390/md20110683)
Supplement: Supplementary file 1 [file marinedrugs-20-00683-s001.zip › marinedrugs-1972377-supplementary.pdf]

Figure S1: Chemical structures of 2－5

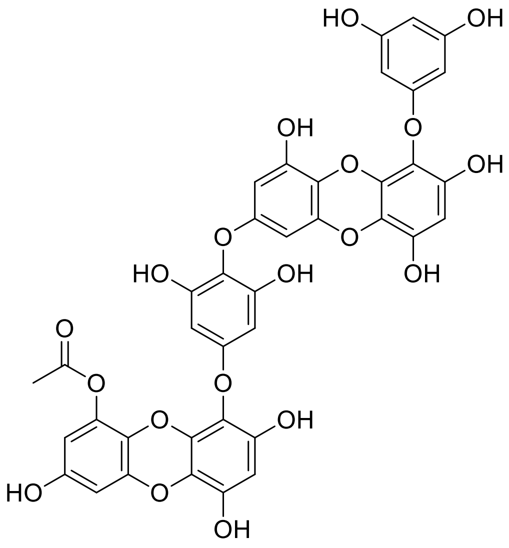

2

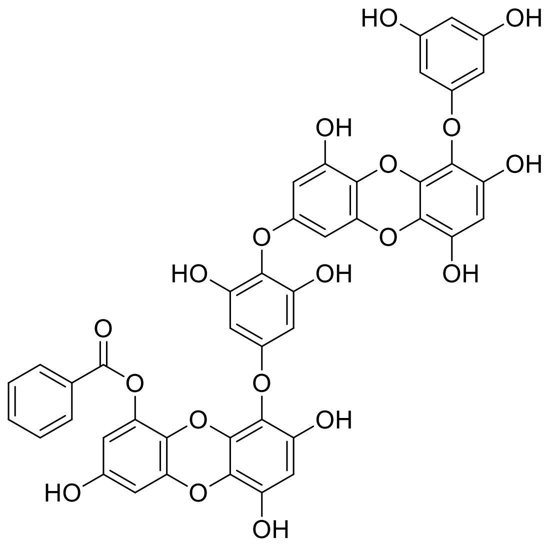

3

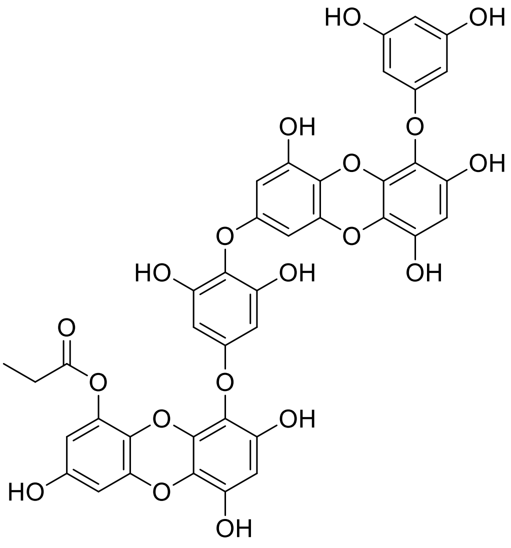

4

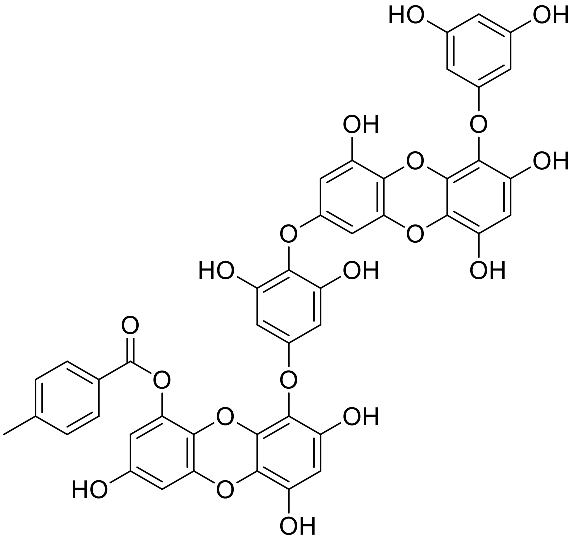

5

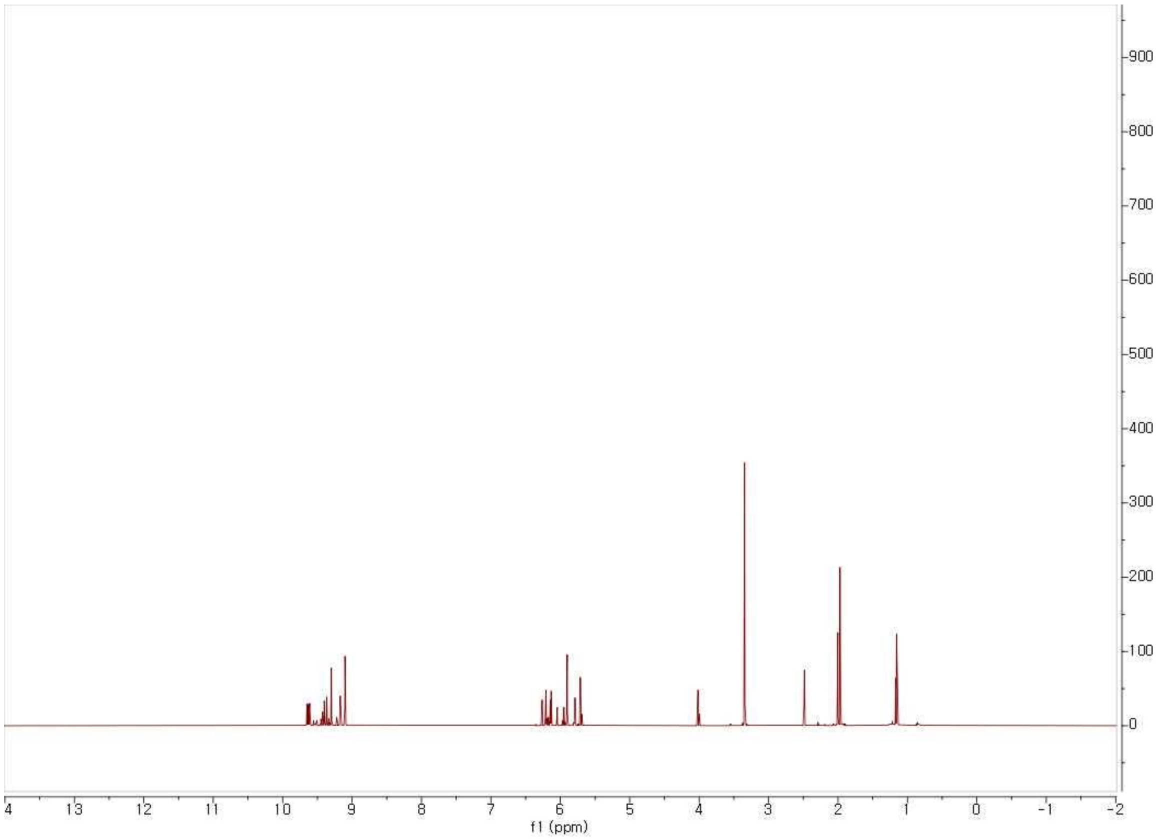

Figure S2. ^{1}H NMR spectrum of **2** (600 MHz, DMSO-d_6)

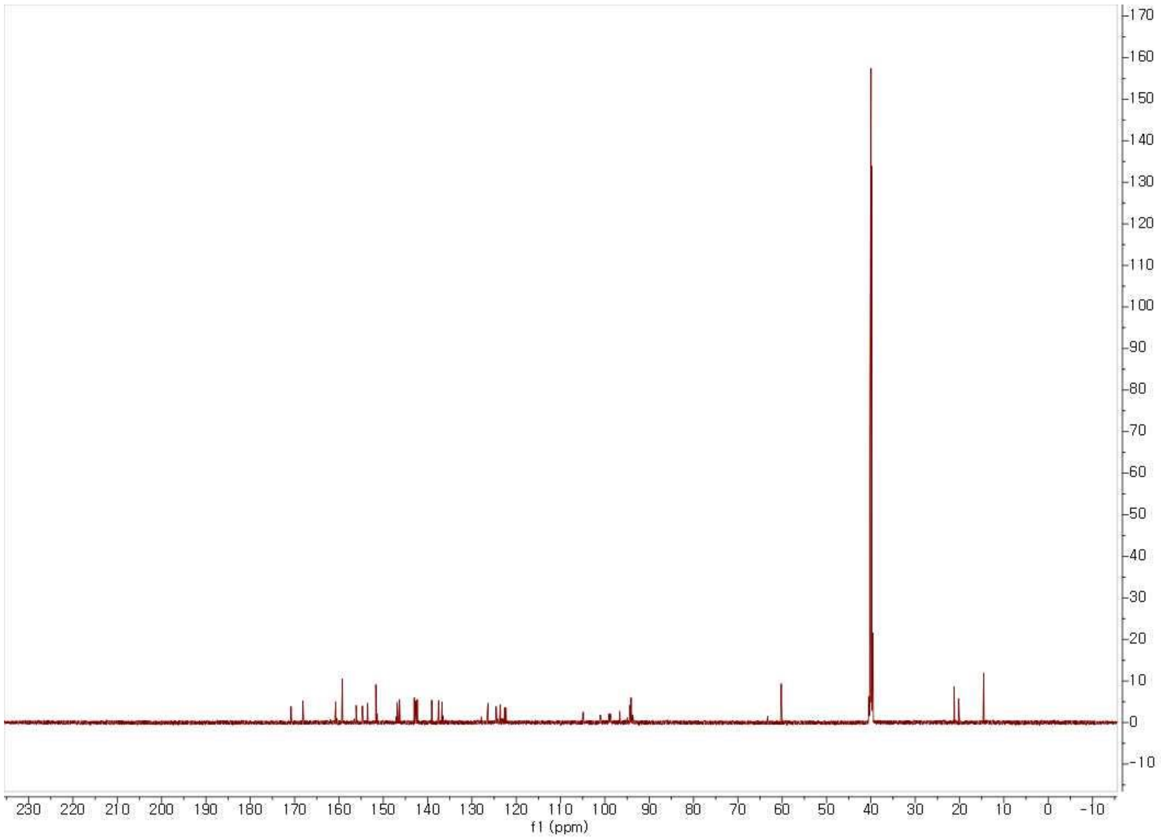

Figure S3. ^{13}C NMR spectrum of **2** (150 MHz, DMSO-d_6)

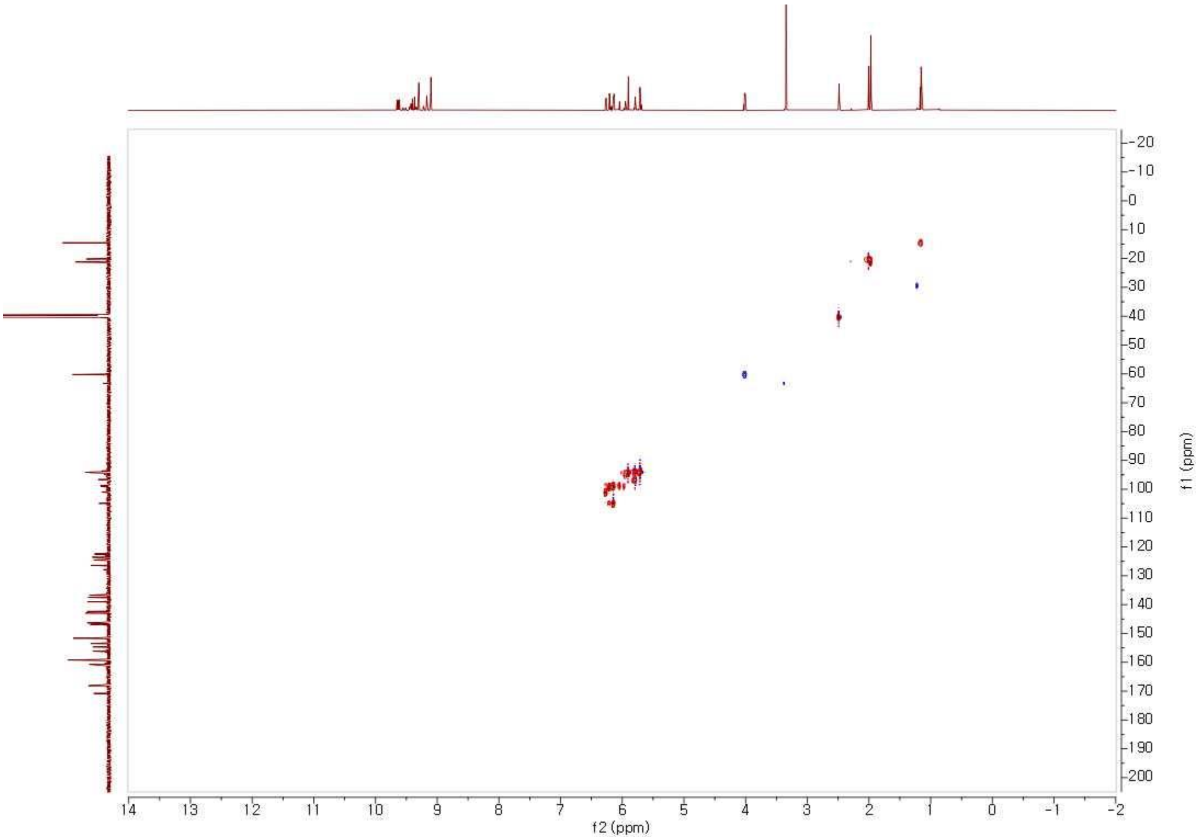

Figure S4. HSQC spectrum of **2** (600 MHz, DMSO-d_6)

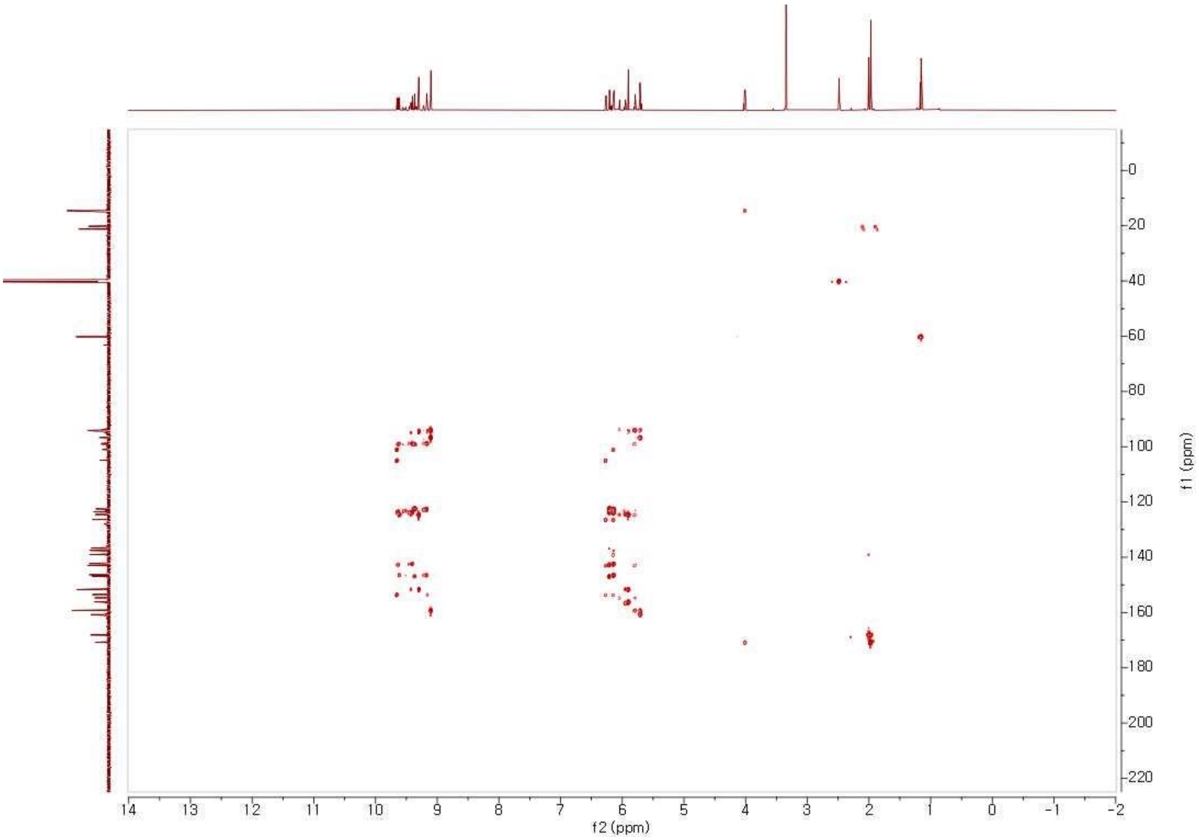

Figure S5. HMBC spectrum of **2** (600 MHz, DMSO-d_6)

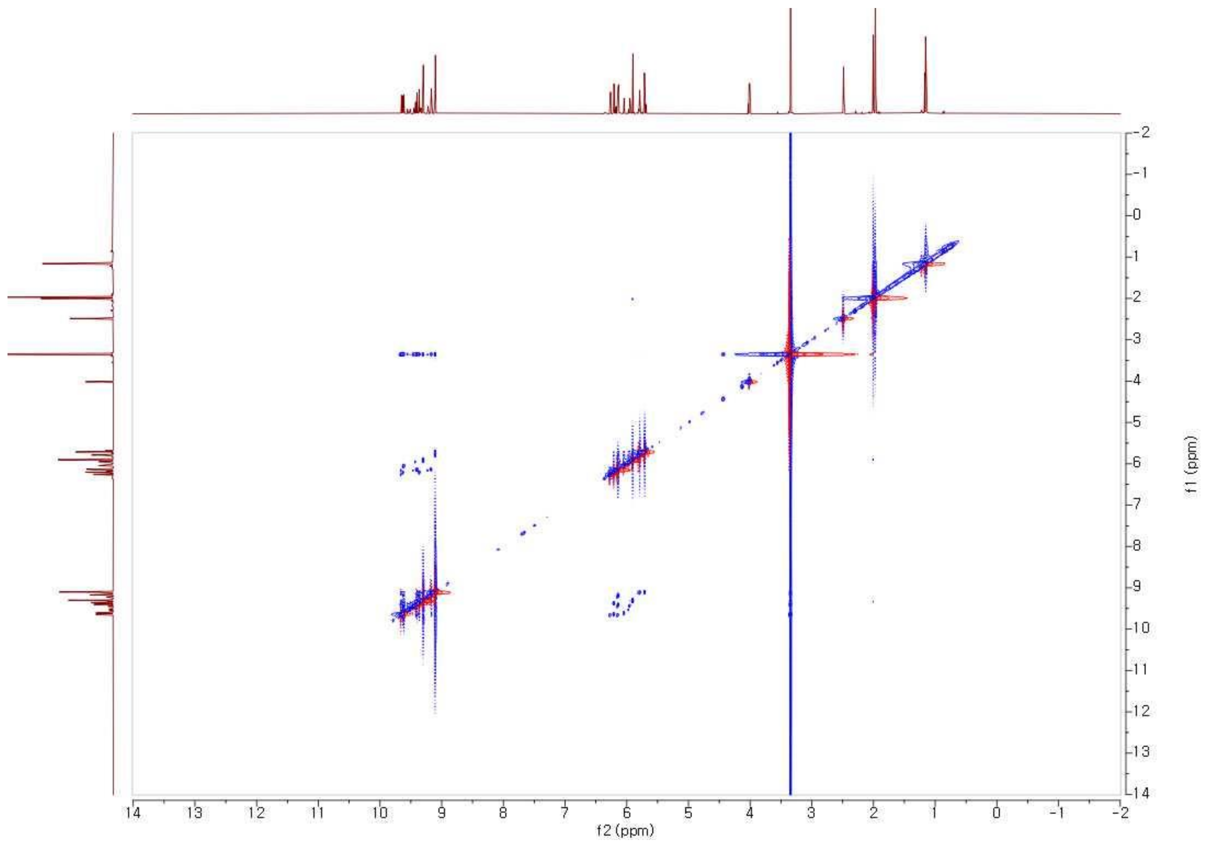

Figure S6. NOESY spectrum of **2** (600 MHz, DMSO-d_6)

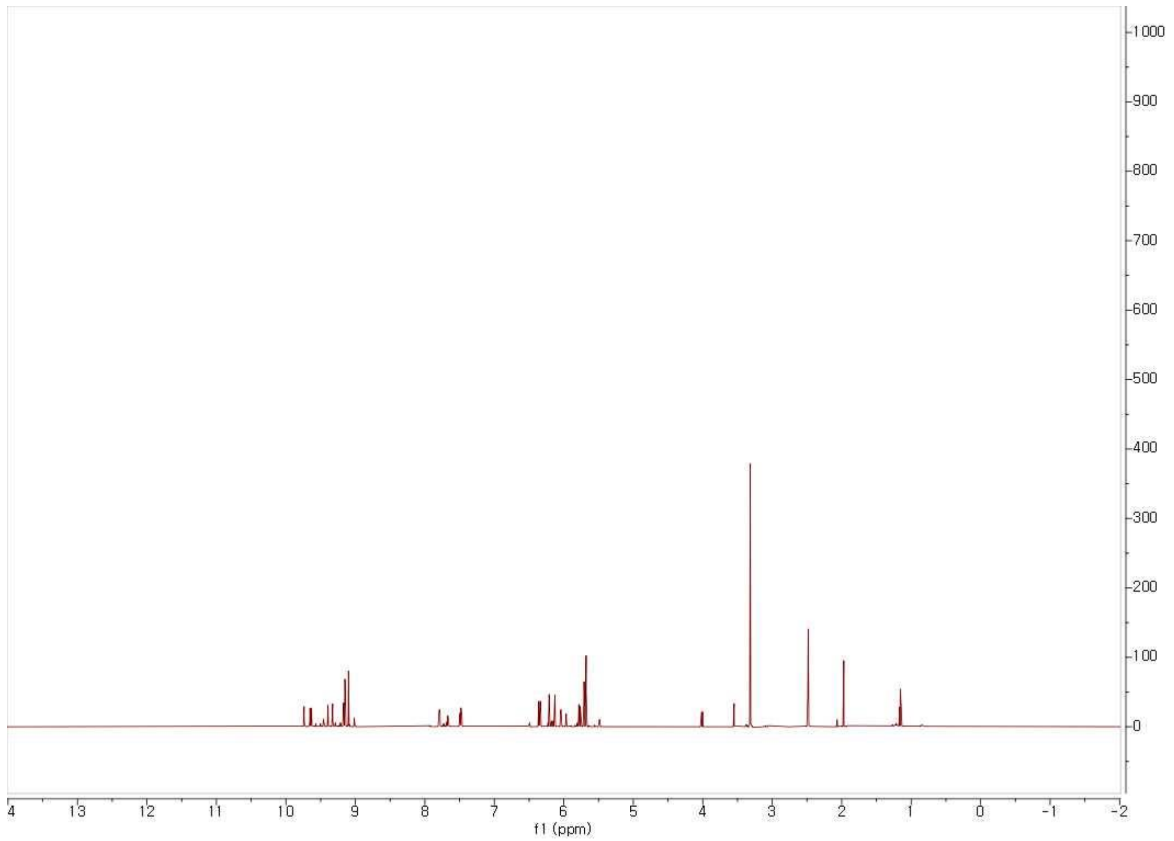

Figure S7. ^{1}H NMR spectrum of **3** (600 MHz, DMSO-d_6)

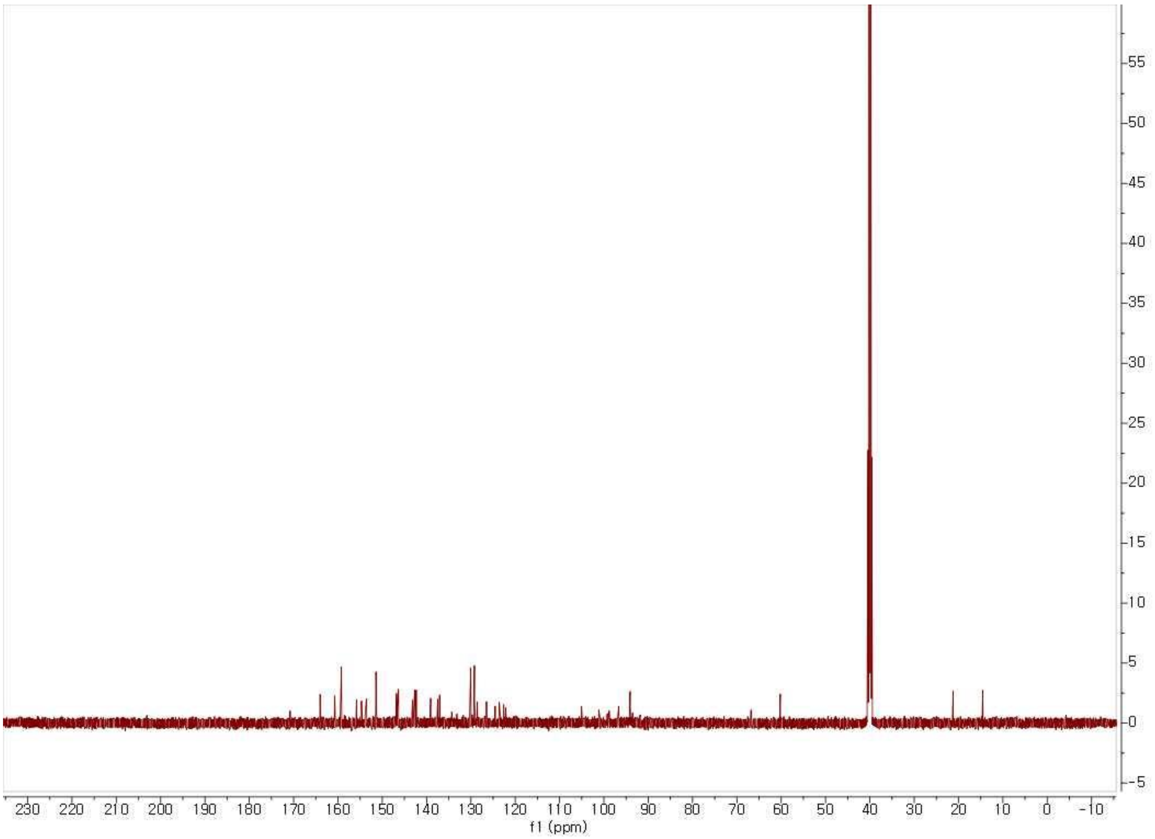

Figure S8. ^{13}C NMR spectrum of **3** (150 MHz, DMSO-d_6)

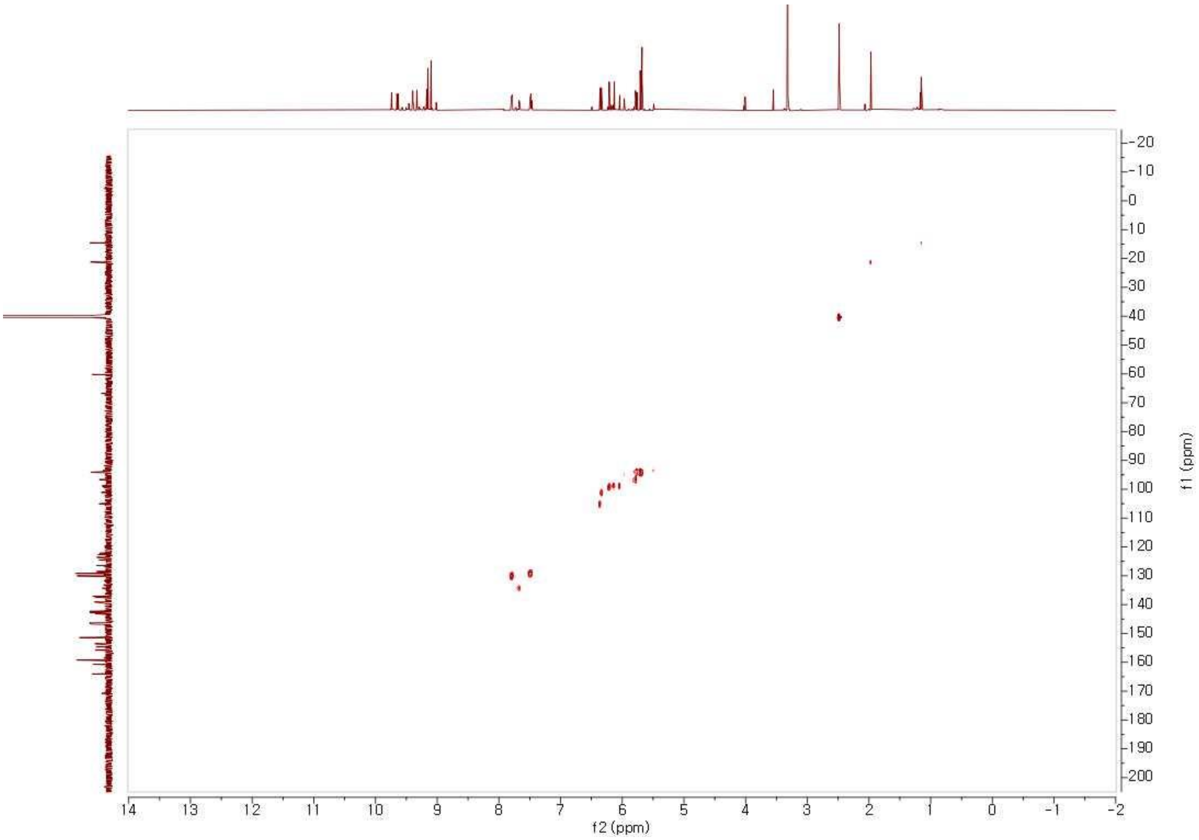

Figure S9. HSQC spectrum of **3** (600 MHz, DMSO-d_6)

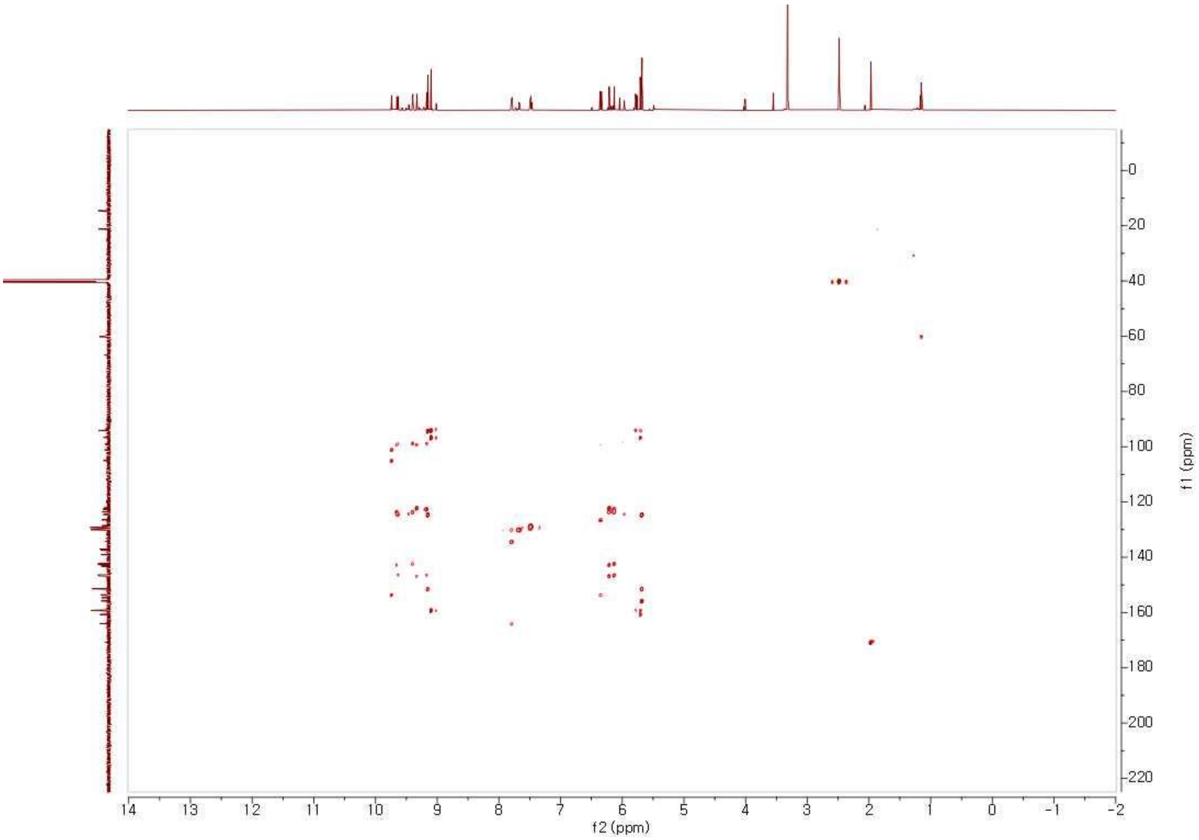

Figure S10. HMBC spectrum of **3** (600 MHz, DMSO-d_6)

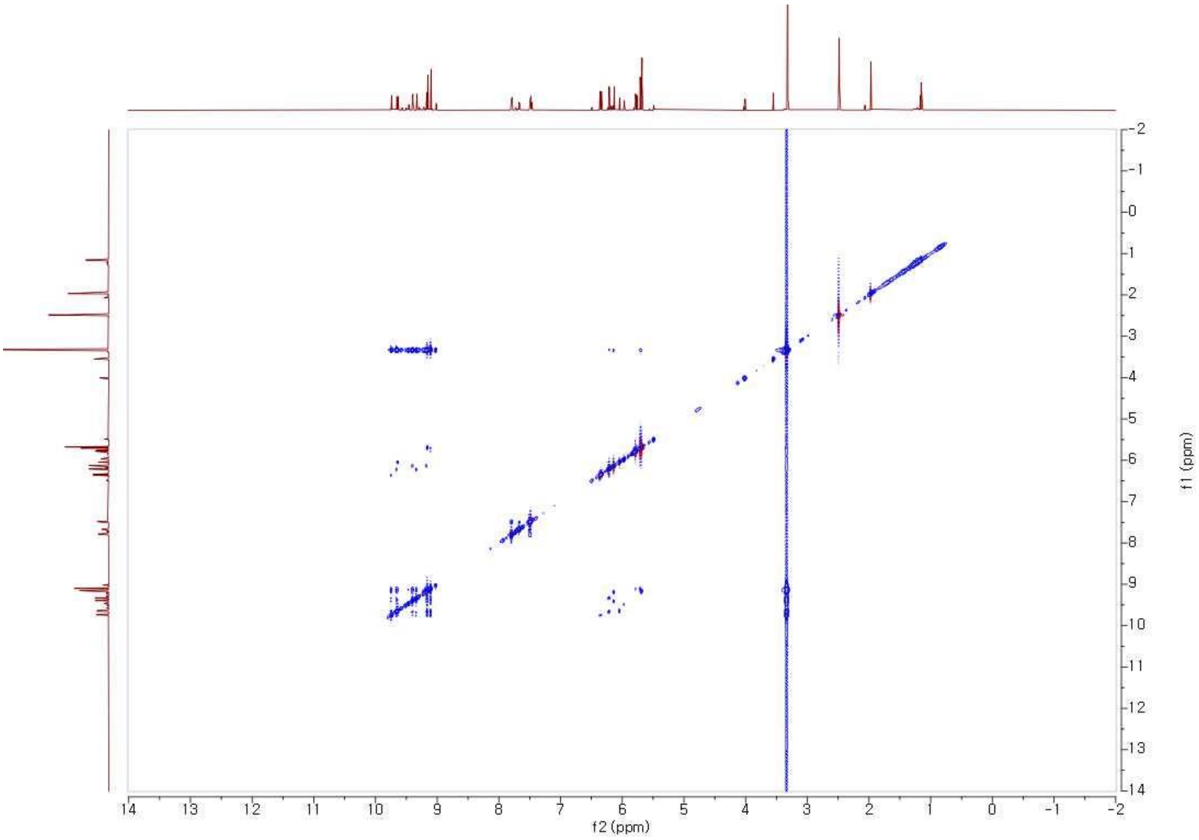

Figure S11. NOESY spectrum of **3** (600 MHz, DMSO-d_6)

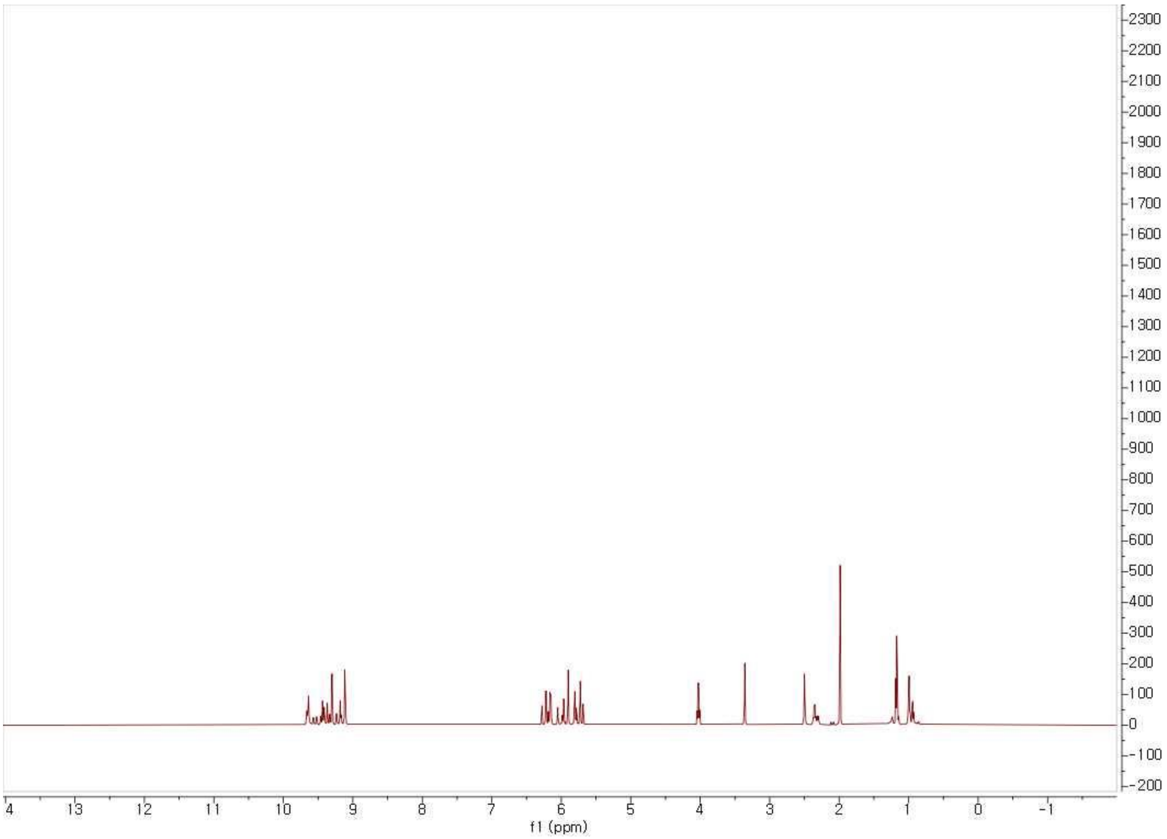

Figure S12. ^{1}H NMR spectrum of **4** (600 MHz, DMSO-d_6)

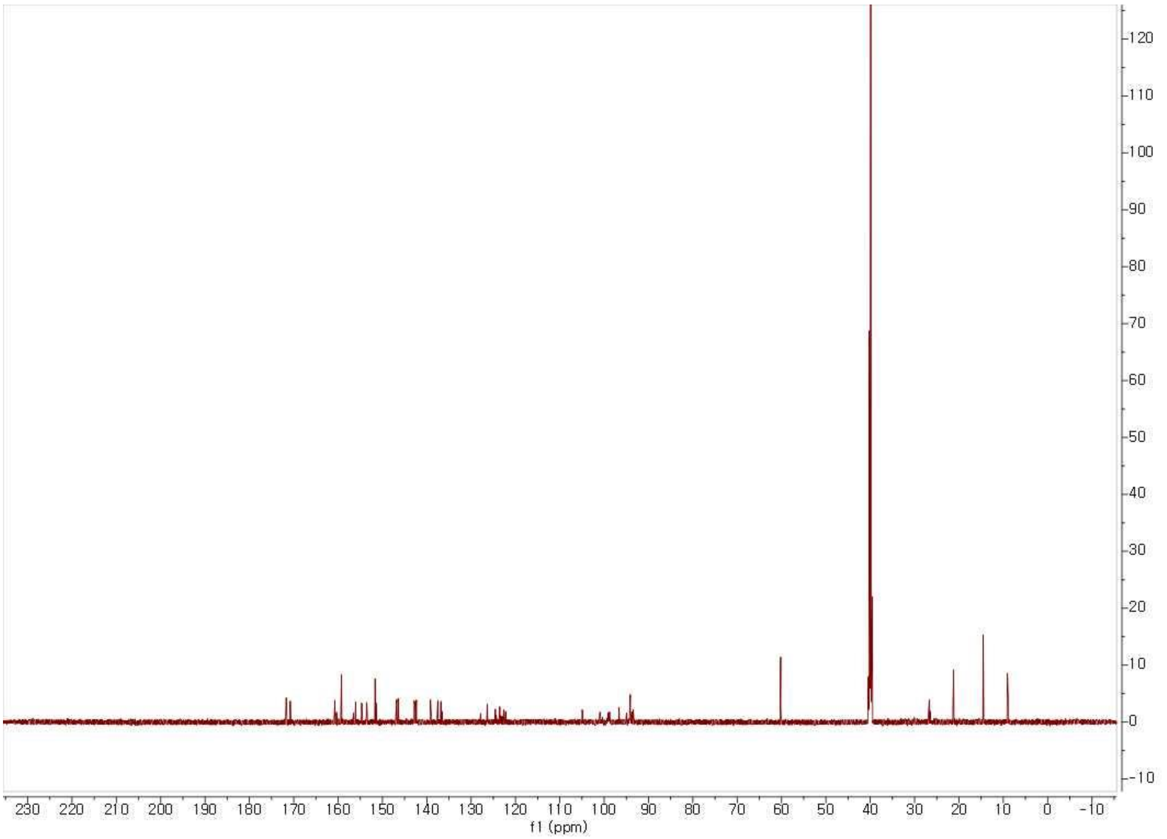

Figure S13. ^{13}C NMR spectrum of **4** (150 MHz, DMSO-d_6)

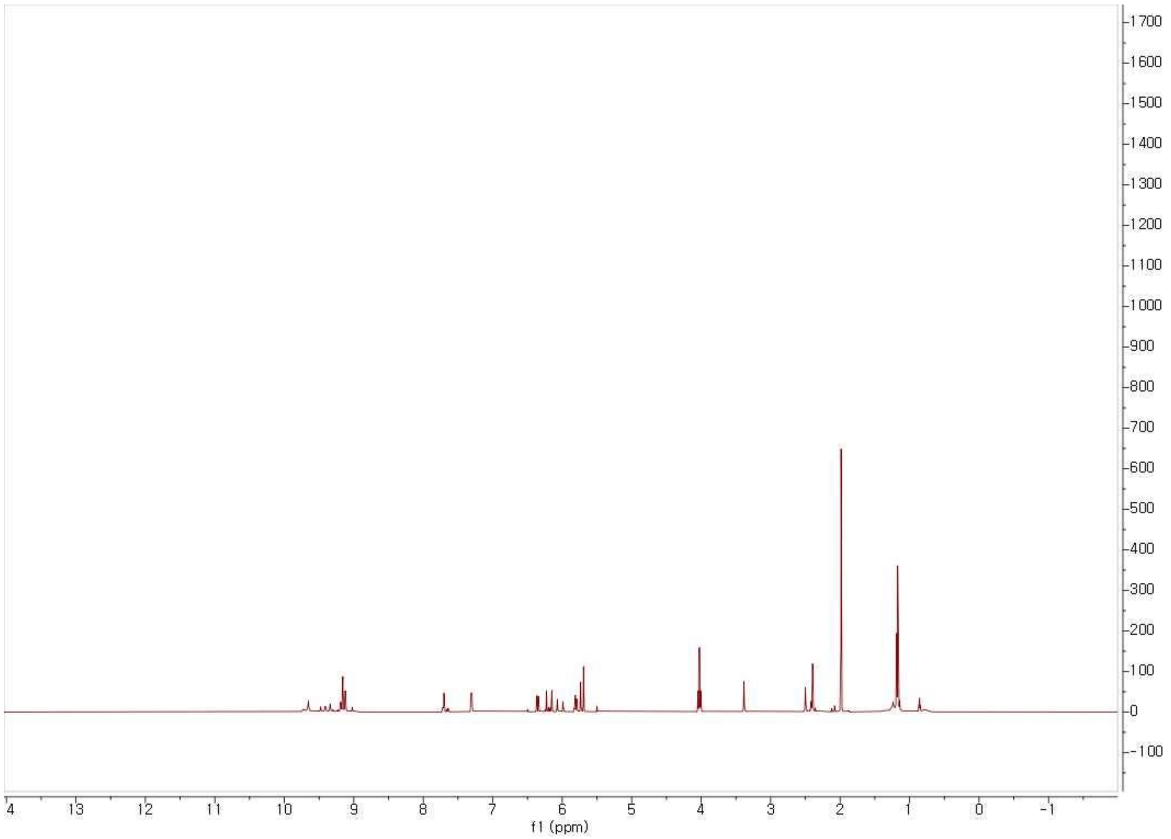

Figure S14. ^{1}H NMR spectrum of **5** (600 MHz, DMSO-d_6)

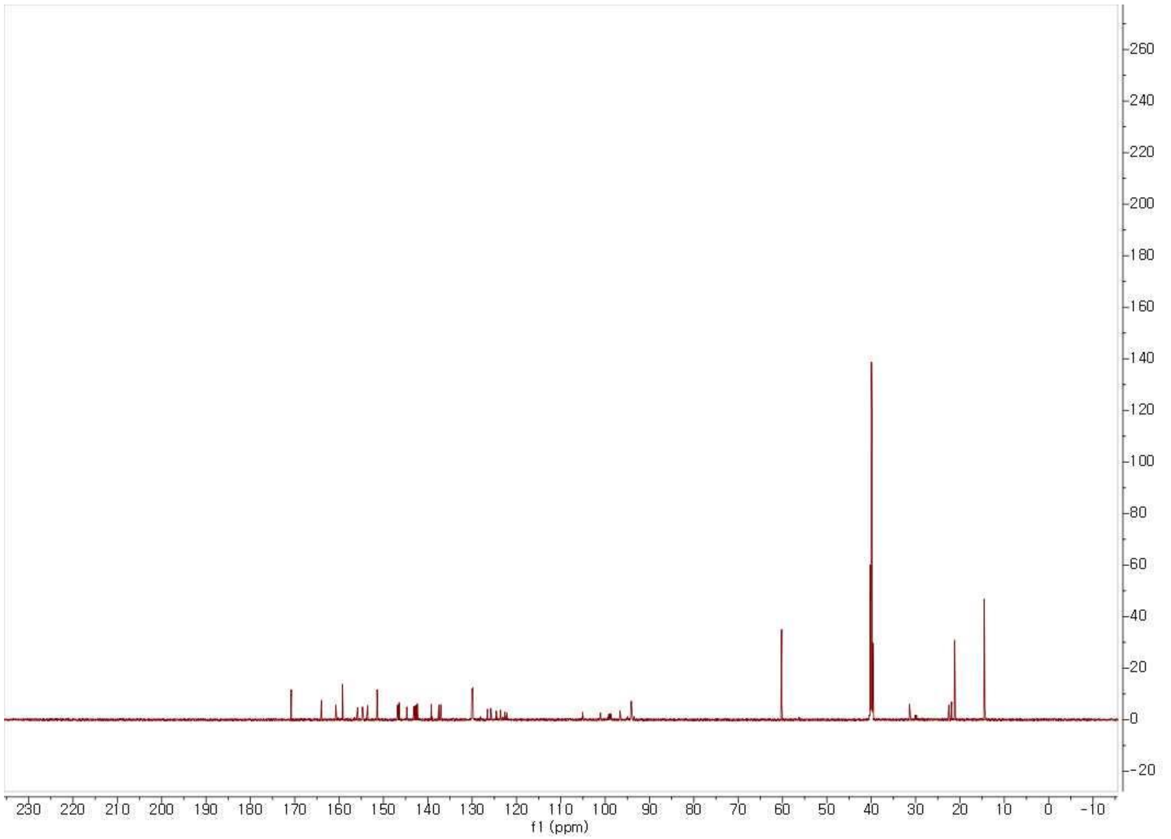

Figure S15. ^{13}C NMR spectrum of **5** (150 MHz, DMSO-d_6)

Table S1. ^{1}H, ^{13}C, HMBC, and NOESY NMR data of **3** (δ in ppm, data obtained in DMSO-d_6).

No.	δ_C	δ_H	(J in Hz)	HMBC (H → C)	NOESY
**1**	142.65	9.67		C_2, C_1, C_{10a}	C_2-H
**2**	99.21	6.23		C_4, C_3, C_1, C_{10a}	C_3-H, C_1-H
**3**	146.76	9.34		C_4, C_3, C_2	C_2-H
**4**	122.14				
**4a**	137.05				
**5a**	126.47				
**6**	139.07				
**7**	105.03	6.38	d (2.73)	C_9, C_8, C_6, C_{5a}	C_8-H
**8**	153.55	9.76		C_9, C_8, C_7	C_9-H, C_7-H
**9**	101.13	6.35	d (2.73)	C_{9a}, C_8, C_7, C_{5a}	C_8-H
**9a**	143.16				
**10a**	123.52				
**1'**	155.79				
**2'**	94.20	5.70		$C_{1'}$, $C_{2'}$, $C_{3'}$, $C_{4'}$, $C_{5'}$, $C_{6'}$	$C_{3'}$-H, $C_{5'}$-H
**3'**	151.41	9.17		$C_{2'}$, $C_{3'}$, $C_{4'}$, $C_{5'}$, $C_{6'}$	$C_{2'}$-H, $C_{6'}$-H
**4'**	124.61				
**5'**	151.41	9.17		$C_{2'}$, $C_{3'}$, $C_{4'}$, $C_{5'}$, $C_{6'}$	$C_{2'}$-H, $C_{6'}$-H
**6'**	94.20	5.70		$C_{1'}$, $C_{2'}$, $C_{3'}$, $C_{4'}$, $C_{5'}$, $C_{6'}$	$C_{3'}$-H, $C_{5'}$-H
**1''**	93.83	5.78	d (2.85)	$C_{10a''}$, $C_{2''}$, $C_{3''}$, $C_{4a''}$	
**2''**	154.66				
**3''**	98.85	6.06	d (2.86)	$C_{1''}$, $C_{2''}$, $C_{4a''}$	$C_{4''}$-H
**4''**	146.33	9.65		$C_{3''}$, $C_{4''}$, $C_{4a''}$	$C_{3''}$-H
**4a''**	124.50				
**5a''**	137.48				
**6''**	122.60				
**7''**	146.46	9.19		$C_{6''}$, $C_{7''}$, $C_{8''}$	$C_{8''}$-H
**8''**	98.73	6.15		$C_{6''}$, $C_{7''}$, $C_{9''}$, $C_{9a''}$	$C_{7''}$-H, $C_{9''}$-H
**9''**	142.31	9.41		$C_{8''}$, $C_{9''}$, $C_{9a''}$	$C_{8''}$-H
**9a''**	123.58				
**10a''**	142.80				
**1'''**	160.70				
**2'''**	94.07	5.73	d (2.09)	$C_{1'''}$, $C_{2'''}$, $C_{3'''}$, $C_{4'''}$, $C_{5'''}$, $C_{6'''}$	$C_{3'''}$-H, $C_{5'''}$-H
**3'''**	159.20	9.12		$C_{2'''}$, $C_{3'''}$, $C_{4'''}$, $C_{5'''}$, $C_{6'''}$	$C_{2'''}$-H,$C_{4'''}$-H, $C_{6'''}$-H
**4'''**	96.62	5.80	t (2.08)	$C_{2'''}$, $C_{3'''}$, $C_{5'''}$, $C_{6'''}$	$C_{3'''}$-H
**5'''**	159.20	9.12		$C_{2'''}$, $C_{3'''}$, $C_{4'''}$, $C_{5'''}$, $C_{6'''}$	$C_{2'''}$-H,$C_{4'''}$-H, $C_{6'''}$-H
**6'''**	94.07	5.73	d (2.09)	$C_{1'''}$, $C_{2'''}$, $C_{3'''}$, $C_{4'''}$, $C_{5'''}$, $C_{6'''}$	$C_{3'''}$-H, $C_{5'''}$-H
**6-O*(CO)*C(CH)$_2$(CH)$_2$(CH)**	164.01				
**6-O(CO)*C*(CH)$_2$(CH)$_2$(CH)**	134.31				
**6-O(CO)C*(CH)$_2$*(CH)$_2$(CH)**	130.02	7.81	dd (7.04, 1.32)	C_6-O*(CO)*C(CH)$_2$(CH)$_2$(CH),	$C_{2'}$-H, $C_{6'}$-H
**6-O(CO)C(CH)$_2$*(CH)$_2$*(CH)**	129.19	7.50	t (7.47)	C_6-O(CO)C*(CH)$_2$*(CH)$_2$(CH)	
**6-O(CO)C(CH)$_2$(CH)$_2$*(CH)***	128.54	7.69	tt (7.47, 1.32)	C_6-O(CO)*C*(CH)$_2$(CH)$_2$(CH)	

Table S2. ^{1}H and ^{13}C NMR data of **4** (δ in ppm, data obtained in DMSO-d_6).

No.	δ_C	δ_H	(J in Hz)
**1**	142.64	9.65	
**2**	99.10	6.22	
**3**	146.80	9.37	
**4**	122.23		
**4a**	136.81		
**5a**	126.37		
**6**	139.16		
**7**	104.95	6.16	d (2.71)
**8**	153.53	9.66	
**9**	100.96	6.28	d (2.71)
**9a**	143.00		
**10a**	123.53		
**1'**	156.04		
**2'**	94.22	5.90	
**3'**	151.65	9.30	
**4'**	124.62		
**5'**	151.65	9.30	
**6'**	94.22	5.90	
**1''**	93.73	5.81	d (2.87)
**2''**	154.67		
**3''**	98.83	6.05	d (2.87)
**4''**	146.34	9.64	
**4a''**	124.52		
**5a''**	137.52		
**6''**	122.63		
**7''**	146.47	9.18	
**8''**	98.74	6.15	
**9''**	142.33	9.44	
**9a''**	123.60		
**10a''**	142.84		
**1'''**	160.72		
**2'''**	94.09	5.73	d (2.11)
**3'''**	159.21	9.12	
**4'''**	96.63	5.80	t (2.11)
**5'''**	159.21	9.12	
**6'''**	94.09	5.73	d (2.11)
**6-O*(CO)*CH_2CH_3**	171.25		
**6-O(CO)*CH_2*CH_3**	26.67	2.36	q (7.48)
**6-O(CO)CH_2*CH_3***	9.07	0.99	t (7.48)

Table S3. ^{1}H and ^{13}C NMR data of **5** (δ in ppm, data obtained in DMSO-d_6).

No.	δ_C	δ_H	(*J* in Hz)
**1**	142.66	9.65	
**2**	99.19	6.23	
**3**	146.78	9.34	
**4**	122.17		
**4a**	137.07		
**5a**	126.51		
**6**	139.19		
**7**	105.07	6.37	d (2.74)
**8**	153.53	9.73	
**9**	101.04	6.34	d (2.74)
**9a**	143.17		
**10a**	123.58		
**1'**	155.81		
**2'**	94.21	5.69	
**3'**	151.43	9.16	
**4'**	124.68		
**5'**	151.43	9.16	
**6'**	94.21	5.69	
**1''**	93.93	5.79	d (2.88)
**2''**	154.70		
**3''**	98.92	6.07	d (2.88)
**4''**	146.34	9.65	
**4a''**	124.54		
**5a''**	137.51		
**6''**	122.64		
**7''**	146.48	9.19	
**8''**	98.75	6.15	
**9''**	142.34	9.41	
**9a''**	123.62		
**10a''**	142.82		
**1'''**	160.73		
**2'''**	94.10	5.73	d (2.10)
**3'''**	159.23	9.12	
**4'''**	96.65	5.81	t (2.10)
**5'''**	159.23	9.12	
**6'''**	94.10	5.73	d (2.10)
**6-O*(CO)*C(CH)$_2$(CH)$_2$CCH$_3$**	163.99		
**6-O(CO)*C*(CH)$_2$(CH)$_2$CCH$_3$**	125.79		
**6-O(CO)C*(CH)$_2$*(CH)$_2$CCH$_3$**	130.08	7.71	d (8.05)
**6-O(CO)C(CH)$_2$*(CH)$_2$*CCH$_3$**	129.84	7.31	d (8.05)
**6-O(CO)C(CH)$_2$(CH)$_2$*C*CH$_3$**	144.75		
**6-O(CO)C(CH)$_2$(CH)$_2$C*CH$_3$***	21.87	2.40	
